# Redescription of *Monacha pantanellii* (De Stefani, 1879), a species endemic to the central Apennines, Italy (Gastropoda, Eupulmonata, Hygromiidae) by an integrative molecular and morphological approach

**DOI:** 10.3897/zookeys.988.56397

**Published:** 2020-11-06

**Authors:** Joanna R. Pieńkowska, Giuseppe Manganelli, Folco Giusti, Debora Barbato, Ewa Kosicka, Alessandro Hallgass, Andrzej Lesicki

**Affiliations:** 1 Department of Cell Biology, Institute of Experimental Biology, Faculty of Biology, Adam Mickiewicz University in Poznan, Uniwersytetu Poznańskiego 6, 61-614 Poznań, Poland Adam Mickiewicz University in Poznan Poznań Poland; 2 Dipartimento di Scienze Fisiche, della Terra e dell’Ambiente, Università di Siena, Via Mattioli 4, 53100 Siena, Italy Università di Siena Siena Italy

**Keywords:** 16S rDNA, COI, H3, ITS2, molecular features, shell and genital structure, species distribution

## Abstract

Specimens obtained from ten populations of a *Monacha* species from the central Apennines were compared with six molecular lineages of *Monacha cantiana* s. l. (CAN-1, CAN-2, CAN-3, CAN-4, CAN-5, CAN-6) and two other *Monacha* species (*M. cartusiana* and *M. parumcincta*), treated as outgroup, by molecular (nucleotide sequences of two mitochondrial COI and 16S rDNA as well as two nuclear ITS2 and H3 gene fragments) and morphological (shell and genital anatomy) analysis. The results strongly suggest that these populations represent a separate species for which two names are available: the older *Helix pantanellii* De Stefani, 1879 and the junior *M. ruffoi* Giusti, 1973. The nucleotide sequences created well separated clades on each phylogenetic tree. Genital anatomy included several distinctive features concerning vaginal appendix, penis, penial papilla and flagellum; instead, shell characters only enabled them to be distinguished from *M. cartusiana* and *M. parumcincta*. Remarkably, populations of *M. pantanellii* show high morphological variability. Shell variability mainly concerns size, some populations having very small dimensions. Genital variability shows a more intricate pattern of all anatomical parts, being higher as regards the vagina and vaginal appendix. Despite this morphological variability, the K2P distance range of COI sequences between populations is narrow (0.2–4.5%), if we consider all but three of the 53 sequences obtained. This research confirmed that the species of *Monacha* and their molecularly distinguished lineages can only occasionally be recognised morphologically and that they have significant inter- and intra-population variability. The possibility of using an overall approach, including shell, genital and molecular evidence, was taken in order to establish a reliable taxonomic setting.

## Introduction

Land snail fauna of the central and southern Apennines of Italy includes many common, widespread and diversified helicoideans, such as the geomitrids *Candidula* Kobelt, 1871 and *Xerogyra* Monterosato, 1892, the hygromiid *Monacha* Fitzinger, 1833, the helicids *Marmorana* Hartmann, 1844 and *Helix* Linnaeus, 1758. Despite this, their taxonomy, systematics and phylogenetics have been challenging since the early studies exclusively based on shell features. Taxonomic revisions of the second half of the 20^th^ century (e.g., Forcart 1965; Giusti 1973) lumped many of the earliest described taxa on the basis of a similar gross genital morphology. However, more recent investigations using protein electrophoresis/allozymes (*Marmorana*: Oliverio et al. 1993) and mitochondrial and nuclear gene sequences (*Marmorana*: Fiorentino et al. 2010; *Helix*: Fiorentino et al. 2016) shed new light on these variable species and radiation may explain the relationship between the lineages or clades distinguished in the Apennines.

Continuing work on the hygromiid *Monacha* (Pieńkowska et al. 2015, 2016, 2018a, 2018b, 2019a, 2019b), we studied species living in the mountain grasslands of the central Apennines, whence came reports of three species, the widespread *M. cantiana* (Montagu, 1803) and the endemic *M. orsini* (Villa & Villa, 1841) and *M. ruffoi* Giusti, 1973, and a number of taxa with uncertain taxonomic status (Alzona 1971; Manganelli et al. 1995). We conducted a joint molecular and morphological study of many populations, finding many different species or their molecular lineages. However, it was difficult to draw reliable nomenclatural and taxonomic conclusions because the identity of the earliest taxa, established in the past, were often based on non-diagnostic shell characters of specimens without any precise collecting record.

A first result of our research corroborated the specific distinctness of *Monacha ruffoi* Giusti, 1973, of which we discovered an overlooked senior synonym: *Helix pantanellii* De Stefani, 1879.

The aim of the present research was: 1) to investigate phylogenetic relationships of *Monacha pantanellii* with other *Monacha* species or their molecular lineages; 2) to evaluate its morphological variability; 3) to redescribe the species.

## Materials and methods

### Taxonomic sampling

**Figure 1.**
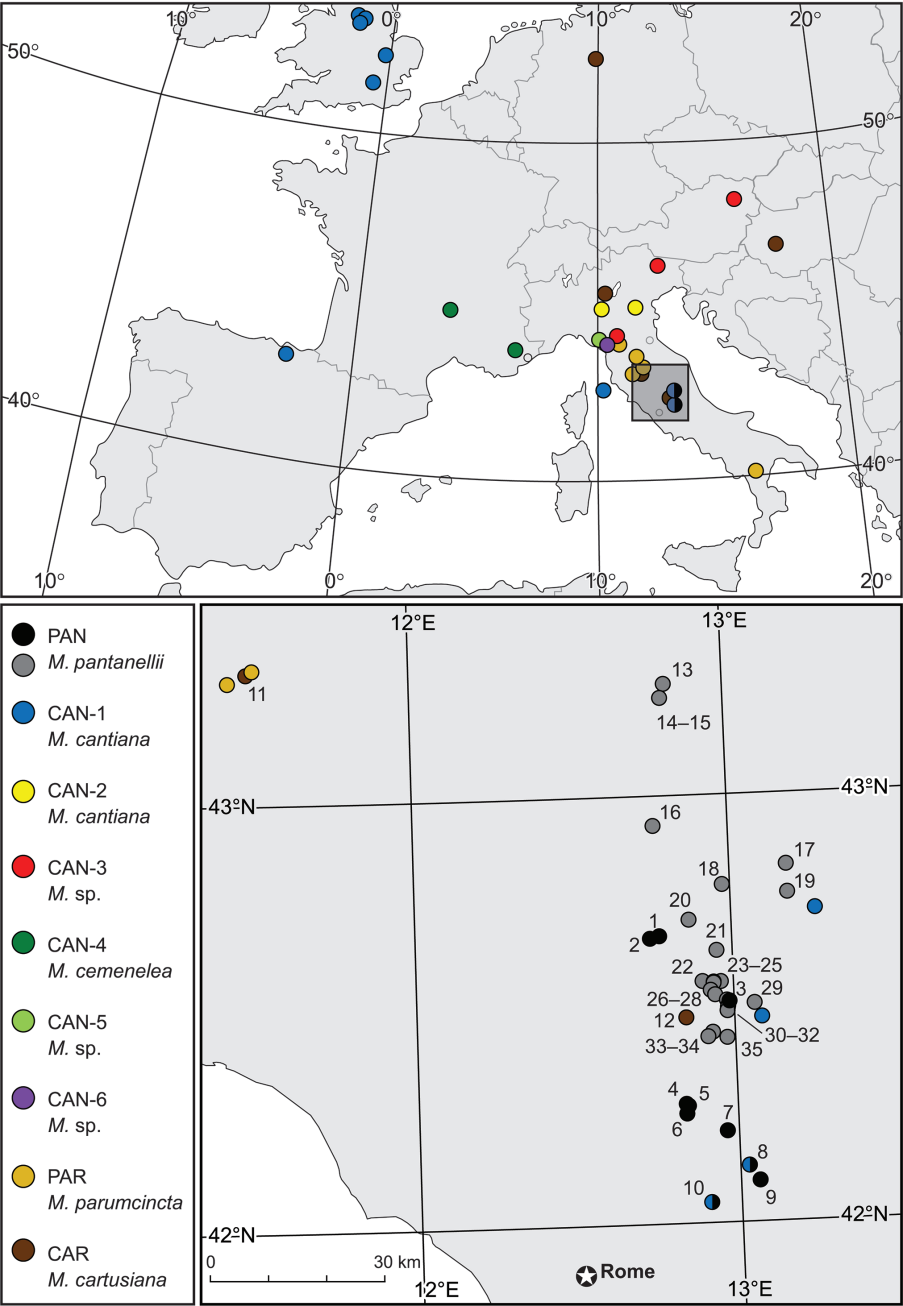
Localities of *Monacha pantanellii* and *M. cartusiana* populations listed in Tables 1, 3 (*M. pantanellii* black circles Table 1, grey circles Table 3). Details of localities of other *Monacha* species and their molecular lineages were provided in previous papers (Pieńkowska et al. 2015, 2018a, 2018b, 2019b).

Ten populations of *Monacha pantanellii* (Table 1, Fig. 1) were considered in our analysis of their molecular and morphological (shell and genitalia structure) variability, and compared with the *M. cantiana* s. l. lineages (Pieńkowska et al. 2018a, 2019b). The sequences deposited in GenBank were also considered for the molecular analysis (Table 2). Two other *Monacha* species were used for morphological and molecular comparison: *M. cartusiana* (Müller, 1774) and *M. parumcincta* (Rossmässler, 1834). Another 23 populations of *M. pantanellii* were studied on a qualitative morphological basis (they were not included in the statistical analysis) (Table 3).

**Table 1.** List of localities of populations of *Monacha pantanellii* used for molecular and morphological research. A question mark before the geographical coordinates of the locality no. 3 denotes that the georeferencing was done a posteriori on the basis of the available information.

No.	Localities			Clade	Popu-lation	COI		16S rDNA		ITS2		H3		Figs
	Coordinates (Lat & Long / UTM references)	Country and site	Collector / date / no. of specimens (collection)			New haplotype (no. spcms.)	GenBank ##	New haplotype (no. spcms.)	GenBank ##	New common sequence (no. spcms)	GenBank ##	New common sequence (no. spcms)	GenBank ##	
1	42°40.35'N, 12°46.29'E 33TUH12	Italy, Umbria, Monte Fionchi, summit (Spoleto, Perugia), 1340 m a.s.l.	G. Manganelli & L. Manganelli / 12.09.1999 / 5 (FGC 8140)	PAN	Fio1	COI 1 (2)	MT380011	16S 1 (3)	MT376031			H3 1 (1)	MT385776	5, 6, 37–40
							MT380012		MT376032			H3 2 (3)	MT385777	
						COI 2 (1)	MT380013		MT376033	ITS2 1 (1)	MT376088		MT385778	
								16S 2 (1)	MT376034	ITS2 2 (1)	MT376089		MT385779	
												H3 3 (1)	MT385780	
2	42°40.05'N, 12°44.53'E 33TUH12	Italy, Umbria, Monte Fionchi, 900 NE di Torrecola (Spoleto, Perugia), 680 m a.s.l.	A. Hallgass / 2010 / 5 (FGC 38944)	PAN	Fio2	COI 3 (1)	MT380014	16S 3 (1)	MT376035	ITS2 2 (2)	MT376090	H3 2 (2)	MT385781	7, 41–44
						COI 4 (1)	MT380015	16S 4 (1)	MT376036		MT376091		MT385782	
						COI 5 (1)	MT380016	16S 5 (1)	MT376037	ITS2 3 (1)	MT376092	H3 4 (1)	MT385783	
						COI 6 (2)	MT380017	16S 2 (2)	MT376038	ITS2 2 (2)	MT376093	H3 5 (1)	MT385784	
							MT380018		MT376039		MT376094	H3 2 (1)	MT385785	
3	? 42°31.13'N, ? 12°58.63'E 33TUH30	Italy, Vallonina (Monti Reatini, Lazio)	F. Giusti / 03.08.1966 / 5 (FGC 10883, 25345)	PAN	Val	COI 7 (1)	MT380019	16S 6 (5)	MT376040			H3 6 (2)	MT385786	21–22, 45–48
									MT376041				MT385787	
									MT376042			H3 2 (1)	MT385788	
									MT376043			H3 7 (1)	MT385789	
						COI 8 (1)	MT380020		MT376044			H3 6 (1)	MT385790	
4	42°16.74'N, 12°50.28'E 33TUG28	Italy, Latium, road to Montenero Sabino, 800 m W of Ornaro Alto (Montenero Sabino, Rieti), 670 m a.s.l.	A. Hallgass / 10.2013 / 5 (FGC 41552)	PAN	Sab	COI 9 (1)	MT380021	16S 7 (1)	MT376045			H3 8 (1)	MT385791	8–10, 63
						COI 10 (1)	MT380022	16S 8 (3)	MT376046	ITS2 4 (1)	MT376095	H3 9 (1)	MT385792	
						COI 11 (1)	MT380023		MT376047	ITS2 5 (1)	MT376096	H3 1 (1)	MT385793	
						COI 12 (1)	MT380024		MT376048	ITS2 6 (2)	MT376097	H3 10 (1)	MT385794	
						COI 13 (1)	MT380025	16S 9 (1)	MT376049		MT376098	H3 9 (1)	MT385795	
5	42°16.51'N, 12°50.70'E 33TUG28	Italy, Latium, Via Salaria, 500 m WSW of Ornaro Alto (Torricella in Sabina, Rieti), 520 m a.s.l.	A. Hallgass / 10.2013 / 6 (FGC 41553)	PAN	Alt	COI 14 (1)	MT380026	16S 8 (1)	MT376050	ITS2 7 (1)	MT376099	H3 2 (1)	MT385796	18–20, 61
						COI 15 (1)	MT380027	16S 10 (4)	MT376051	ITS2 3 (1)	MT376100	H3 9 (1)	MT385797	
						COI 16 (2)	MT380028		MT376052	ITS2 7 (1)	MT376101	H3 11 (1)	MT385798	
							MT380029		MT376053	ITS2 8 (1)	MT376102	H3 9 (1)	MT385799	
						COI 17 (1)	MT380030		MT376054			H3 6 (1)	MT385800	
						COI 18 (1)	MT380031	16S 11 (1)	MT376055	ITS2 5 (1)	MT376103	H3 12 (1)	MT385801	
6	42°15.38'N, 12°50.32'E 33TUG28	Italy, Latium, Via Salaria, 650 m NW of Poggio San Lorenzo (Poggio San Lorenzo, Rieti), 400 m a.s.l.	A. Hallgass / 10.2013 / 6 (FGC 41551)	PAN	Lor	COI 19 (1)	MT380032	16S 8 (2)	MT376056	ITS2 9 (1)	MT376104	H3 6 (1)	MT385802	23–25
						COI 20 (1)	MT380033		MT376057	ITS2 5 (2)	MT376105	H3 9 (1)	MT385803	
						COI 21 (4)	MT380034	16S 10 (4)	MT376058		MT376106	H3 1 (1)	MT385804	
							MT380035		MT376059	ITS2 9 (1)	MT376107	H3 11 (1)	MT385805	
							MT380036		MT376060	ITS2 3 (1)	MT376108	H3 1 (1)	MT385806	
							MT380037		MT376061	ITS2 9 (1)	MT376109	H3 6 (1)	MT385807	
7	42°12.81'N, 12°57.80'E 33TUG37	Italy, Latium, near the bridge on Lago del Turano (Castel di Tora, Rieti), 260 m a.s.l.	A. Hallgass / 04.11.2013 / 7 (FGC 41654)	PAN	Tur2	COI 22 (1)	MT380038	16S 12 (2)	MT376062	ITS2 10 (1)	MT376110	H3 13 (1)	MT385808	15–17, 57–59
						COI 23 (1)	MT380039		MT376063			H3 14 (1)	MT385809	
						COI 24 (3)	MT380040	16S 13 (3)	MT376064	ITS2 2 (2)	MT376111	H3 1 (2)	MT385810	
							MT380041		MT376065		MT376112		MT385811	
							MT380042		MT376066	ITS2 5 (1)	MT376113	H3 9 (2)	MT385812	
						COI 25 (1)	MT380043	16S 14 (1)	MT376067	ITS2 11 (1)	MT376114		MT385813	
						COI 26 (1)	MT380044	16S 15 (1)	MT376068	ITS2 5 (1)	MT376115	H3 1 (1)	MT385814	
8	42°07.88'N, 13°01.67'E 33TUG36	Italy, Latium, Valle del Turano, 1,6 km ESE di Turania (Turania, Rieti), 570 m a.s.l.	A. Hallgass / 04.11.2013 / 7 (FGC 42971)	PAN	Tur1	COI 27 (3)	MT380045	16S 16 (3)	MT376069	ITS2 12 (1)	MT376116	H3 10 (1)	MT385815	11–14, 62
							MT380046		MT376070	ITS2 11 (1)	MT376117	H3 9 (2)	MT385816	
							MT380047		MT376071				MT385817	
						COI 28 (1)	MT380048	16S 17 (1)	MT376072	ITS2 2 (1)	MT376118	H3 1 (3)	MT385818	
						COI 29 (1)	MT380049	16S 18 (1)	MT376073	ITS2 11 (3)	MT376119		MT385819	
						COI 22 (1)	MT380050	16S 12 (1)	MT376074		MT376120		MT385820	
						COI 30 (1)	MT380051	16S 19 (1)	MT376075		MT376121	H3 9 (1)	MT385821	
9	42°05.74'N, 13°03.56'E 33TUG36	Italy, Abruzzi, Carsoli, industrial area (Carsoli, L’Aquila), 600 m a.s.l.	A. Hallgass / 04.11.2013 / 5 (FGC 41651)	PAN	Car	COI 19 (1)	MT380052	16S 8 (1)	MT376076			H3 9 (4)	MT385822	30–31, 53–56
						COI 31 (1)	MT380053	16S 20 (1)	MT376077	ITS2 11 (1)	MT376122		MT385823	
						COI 32 (1)	MT380054	16S 21 (1)	MT376078				MT385824	
						COI 8 (2)	MT380055	16S 22 (1)	MT376079	ITS2 5 (2)	MT376123			
							MT380056	16S 19 (1)	MT376080		MT376124		MT385825	
10	42°02.85'N, 12°54.33'E 33TUG25	Italy, Latium, Valle dell’Aniene, 600 m ESE of Roccagiovine (Roccagiovine, Rome), 380 m a.s.l.	A. Hallgass / 10.2013 / 7 (FGC 42974)	PAN	Ani	COI 33 (2)	MT380057	16S 23 (2)	MT376081	ITS2 5 (2)	MT376125	H3 6 (1)	MT385826	26–29, 49–52, 60
							MT380058		MT376082			H3 9 (1)	MT385827	
						COI 34 (1)	MT380059	16S 16 (1)	MT376083		MT376126	H3 1 (1)	MT385828	
						COI 35 (1)	MT380060	16S 24 (1)	MT376084	ITS2 13 (1)	MT376127	H3 15 (1)	MT385829	
						COI 36 (3)	MT380061	16S 25 (3)	MT376085	ITS2 14 (1)	MT376128	H3 9 (1)	MT385830	
							MT380062		MT376086	ITS2 15 (1)	MT376129	H3 14 (1)	MT385831	
							MT380063		MT376087	ITS2 7 (1)	MT376130	H3 9 (1)	MT385832	

### Material examined

New material examined is listed as follows, when possible: geographic coordinates (Lat & Long and UTM references) of locality, locality (country, region, site, municipality and province), collector(s), date, number of specimens (sh/s shell/shells; spcm/spcms specimen/specimens), and collection where material is kept in parenthesis (Tables 1, 3). The material is kept in the F. Giusti collection (**FGC**; Dipartimento di Scienze Fisiche, della Terra e dell’Ambiente, Università di Siena, Italy). The material used for comparison has already been described (see Pieńkowska et al. 2018a: table 1, 2019b: table 1).

### Molecular study

Fifty-eight specimens representing ten population of *Monacha pantanellii* were used for molecular analysis (Table 1). DNA extraction, amplification and sequencing methods are described in detail in our previous paper (Pieńkowska et al. 2018a).

Two mitochondrial and two nuclear gene fragments were analysed, namely cytochrome c oxidase subunit 1 (COI), 16S ribosomal DNA (16S rDNA), an internal transcribed spacer of rDNA (ITS2) and histone 3 (H3), respectively. All new sequences were deposited in GenBank (Table 1). The COI, 16S rDNA, ITS2 and H3 sequences obtained from GenBank for comparison are listed in Table 2.

**Table 2.** GenBank sequences used for molecular analysis comparisons.

Species	COI	16S rDNA	ITS2	H3	References
CAN-1 (*Monacha cantiana* s. s.)	MG208884–MG208924	MG208960–MG208995	MH137963–MH137978	MG209031–MG209039	Pieńkowska et al. (2018a)
	–	–	–	MG209041–MG209048	Pieńkowska et al. (2018a)
CAN-2 (*Monacha cantiana* s. s.)	MG208925–MG208932	MG208996–MG209004	MH137979–MH137981	MG209049–MG209052	Pieńkowska et al. (2018a)
	–	–	MK067000	–	Pieńkowska et al. (2019b)
CAN-3 (*Monacha* sp.)	MG208933–MG208938	MG209005–MG209010	MH137982–MH137983	MG209040	Pieńkowska et al. (2018a)
	–	–	–	MG209053–MG209057	Pieńkowska et al. (2018a)
	–	–	MK067001–MK067002	–	Pieńkowska et al. (2019b)
CAN-4 (*Monacha cemenelea*)	MG208939–MG208943	MG209011–MG209015	MH137984	MG209058–MG209060	Pieńkowska et al. (2018a)
	–	–	MK067003–MK067004	–	Pieńkowska et al. (2019b)
CAN-5 (*Monacha* sp.)	MK066929–MK066941	MK066947–MK066959	MK066981–MK066994	MK066965–MK066977	Pieńkowska et al. (2019b)
CAN-6 (*Monacha* sp.)	MK066942–MK066946	MK066960–MK066964	MK066995–MK066999	MK066978–MK066980	Pieńkowska et al. (2019b)
PAR (*Monacha parumcincta*)	MG208944–MG208959	MG209016–MG209030	MH137985–MH137992	MG209061–MG209071	Pieńkowska et al. (2018a)
	–	–	MK067005	–	Pieńkowska et al. (2019b)
CAR (*Monacha cartusiana*)	KM247376	KM247391	–	–	Pieńkowska et al. (2015)
	–	–	MH137993	MG209072	Pieńkowska et al. (2018a)
	MH203998	MH204081	–	–	Pieńkowska et al. (2018b)

**Table 3.** Populations and materials of *Monacha cartusiana* (CAR) and *Monacha pantanellii* (PAN) not listed in Table 1 because they were not included in the molecular and statistical morphological analysis (apart from additional morphological analysis of *M. cartusiana*). A question mark before the geographical coordinates of some localities denotes that the georeferencing was done a posteriori on the basis of the available information.

No.	Species	Coordinates (Lat & Long / UTM references)	Country and site (municipality and province in parenthesis)	Collector / Date / No. of specimens (collection)	Remarks
11	CAR	43°18.45'N, 11°28.88'E 32TQN09	Italy, Tuscany, Stazione di Castelnuovo Berardenga (Asciano, Siena)	G. Manganelli / 01.11.1981 / spcm (FGC 3430)	–
12	CAR	? 42°28.85'N, 12°50.84'E 33TUH20	Italy, Latium, Lago Lungo (Rieti, Rieti)	F. Giusti / 14.08.1966 / spcm (FGC 23875)	–
13	PAN	? 43°15.67'N, 12°48.83'E 33TUH29	Italy, Umbria, Val Sorda (Gualdo Tadino, Perugia), 1,050 m a.s.l.	A. Minelli / 03.08.1969 / 6 spcms (FGC 25350)	–
14	PAN	? 43°13.72'N, 12°48.02'E 33TUH28	Italy, Umbria, Gualdo Tadino (Gualdo Tadino, Perugia)	F. Giusti / 26.10.1967 / 4 shs (FGC636); 2 spcms (FGC 25352)	–
15	PAN	43°13.72'N, 12°48.02'E 33TUH28	Italy, Umbria, La Rocchetta (Gualdo Tadino, Perugia)	F. Giusti & G. Manganelli / 13.12.1984 / 1 spcm (FGC 6371) / L. Favilli & G. Manganelli / 01.10.1992 / 4 spcms (FGC 6370)	–
16	PAN	42°55.83'N, 12°45.83'E 33TUH15	Italy, Umbria, 600 m a E di Roviglieto (Foligno, Perugia), 510 m a.s.l.	A. Hallgass / 25.09.2010 /	–
17	PAN	42°49.92'N, 13°10.87'E 33TUH54	Italy, Umbria, Monti Sibillini, Valle Canatra (Norcia, Perugia)	F. Giusti & G. Manganelli / 13.09.1988 / 6 shs and 3 spcms (FG 25360)	–
18	PAN	42°47.33'N, 12°58.55'E 33TUH33	Italy, Umbria, Gole di Biselli (Norcia, Perugia)	A. Hallgass / 07.10.2011 /	–
19	PAN	42°46.00'N, 13°10.90'E 33TUH53	Italy, Umbria, Monti Sibillini, Costa Precino (Norcia, Perugia), 1,500 m a.s.l.	A. Benocci, M. Bianchi & G. Manganelli / 29.06.2014 / 2 spcms (FGC 42293)	–
20	PAN	42°42.52'N, 12°51.98'E 33TUH23	Italy, Umbria, 750 m E of Caso (Sant’Anatolia di Narco, Perugia), 800 m a.s.l.	A. Hallgass / 18.09.2010 /	–
21	PAN	42°38.14'N, 12°57.04'E 33TUH32	Italy, Umbria, 1 km SSW of Ruscio (Monteleone di Spoleto, Perugia)	A. Vannozzi / 22.08.2010 /	–
22	PAN	42°33.85'N, 12°54.17'E 3TUH21	Italy, Latium, Monti Reatini, Strada regionale 521 di Morro (Leonessa, Rieti), 1,050 m a.s.l.	A. Hallgass / 13.09.2009 /	–
23	PAN	? 42°33.75'N, 12°57.63'E 33TUH31	Italy, Latium, Monti Reatini, Leonessa (Leonessa, Rieti), 1,000 m a.s.l.	F. Giusti / 04.08.1966 / 1 sh and 1 spcm (FGC 25348)	Paratypes of *Monacha ruffoi* Giusti, 1973
24	PAN	? 42°33.72'N, 12°56.27'E 33TUH31	Italy, Latium, Monti Reatini, Monte Tilia (Leonessa, Rieti), 1,600 m a.s.l.	F. Giusti / 06.08.1966 / 11 shs and 3 spcms (FGC 25337)	Material collected by F. Giusti in 1966 in part published (3 spcms) and in part not published (11 shs). Unfortunately the 3 spcms, constituting paratypes of *Monacha ruffoi* Giusti, 1973, have been lost.
25	PAN	? 42°33.58'N, 12°56.25'E 33TUH31	Italy, Latium, Monti Reatini, Monte Tilia (Leonessa, Rieti), 1,600-1,700 m a.s.l.	F. Giusti / 12.08.1966 / 3 shs (FGC 25338)	Material collected by F. Giusti in 1966 but not published.
26	PAN	? 42°32.58'N, 12°55.65'E 33TUH21	Italy, Latium, Monti Reatini, Monte Corno (Leonessa, Rieti), 1,600 m a.s.l.	F. Giusti / 12.08.1966 / 6 spcms, 2 of which dissected (FGC 25342) / 16 shs (FGC 25340) / 5 shs (FGC 25341)	Paratypes of *Monacha ruffoi* Giusti, 1973
27	PAN	? 42°31.93'N, 12°56.45'E 33TUH31	Italy, Latium, Monti Reatini, Rio Fuggio (Leonessa, Rieti), 1,300 m a.s.l.	F. Giusti / 05.08.1966 / 5 spcms (FGC 25351)	Paratypes of *Monacha ruffoi* Giusti, 1973
28	PAN	? 42°31.13'N, 12°58.63'E 33TUH30	Italy, Latium, Monti Reatini, Vallonina (Leonessa, Rieti), 1,100 m a.s.l.	F. Giusti / 03.08.1966 / 1 spcm (FGC 25343) / 12 shs and 21 spcms (FGC 25344) / 21 spcms, 3 of which dissected (FGC 25345)	Holotype (FGC 25343) and paratypes (FGC 25344 and 25345) of *Monacha ruffoi* Giusti, 1973. Other 5 paratypes from this site have been subject to molecular and morphological study (see Table 1, no. 3)
			Italy, Latium, Monti Reatini, Vallonina (Leonessa, Rieti), 1,100 m a.s.l., along Fiume Corno	F. Giusti / 03.08.1966 / 5 shs (FGC 25347)	Material collected by F. Giusti in 1966 but not published.
29	PAN	? 42°30.63'N, 13°03.85'E 33TUH40	Italy, Latium, Monti Reatini, Monte Cavalli (Posta, Rieti)	F. Giusti / 15.08.1966 / 1 spcm dissected and drawn (FGC)	Material collected by F. Giusti in 1966 but not published and lost.
30	PAN	? 42°30.30'N, 12°58.82'E 33TUH30	Italy, Latium, Monti Reatini, pathway to Monte Sassetelli (Cantalice, Rieti), 1,500 m a.s.l.	F. Giusti / 13.08.1966 / 3 spcms (FGC 25355)	Paratypes of *Monacha ruffoi* Giusti, 1973
31	PAN	? 42°30.12'N, 12°58.77'E 33TUH30	Italy, Latium, Monti Reatini, pathway to Monte Sassetelli (Cantalice, Rieti), 1,550 m a.s.l.	F. Giusti / 13.08.1966 / 3 spcms (FGC 25354)	Material collected by F. Giusti in 1966 but not published.
32	PAN	? 42°29.63'N, 12°58.67'E 33TUH30	Italy, Latium, Monti Reatini, pathway to Monte Sassetelli (Cantalice, Rieti), 1,550-1,750 m a.s.l.	F. Giusti / 13.08.1966 / 3 shs (FGC 25349)	Material collected by F. Giusti in 1966 but not published.
33	PAN	? 42°26.70'N, 12°55.77'E 33TUH20	Italy, Latium, Monti Reatini, above Lisciano (Rieti, Rieti), 800 m a.s.l.	F. Giusti / 6.08.1966 / 14 shs and 2 spcms, 1 of which dissected and drawn (FGC 10890)	Paratypes of *Monacha ruffoi* Giusti, 1973; dissected specimen lost
34	PAN	? 42°26.09'N, 12°54.86'E 33TUH20	Italy, Latium, Monti Reatini, Vazia (Rieti, Rieti), 400 m a.s.l.	F. Giusti / 11.08.1966 / 1 spcm dissected and drawn (FGC)	Material collected by F. Giusti in 1966 but not published and lost.
35	PAN	? 42°25.90'N, 12°58.45'E 33TUG39	Italy, Latium, Monti Reatini, Pian di Stura (Cittaducale, Rieti)	F. Giusti / 07.08.1966 / 1 spcm dissected and drawn (FGC)	Material collected by F. Giusti in 1966 but not published and lost.

The sequences were edited by eye using the programme BioEdit, version 7.2.6 (Hall 1999, BioEdit 2017). Alignments were performed using CLUSTALW (Thompson et al. 1994) implemented in MEGA7 (Kumar et al. 2016). The COI and H3 sequences were aligned according to the translated amino acid sequences. The ends of all sequences were trimmed. The lengths of the sequences after trimming were 592 bp for COI, 286 positions for 16S rDNA, 501 positions for ITS2 and 279 bp for H3. The sequences were collapsed to haplotypes (COI and 16S rDNA) and to common sequences (ITS2 and H3) using the programme ALTER (Alignment Transformation EnviRonment) (Glez-Peña et al. 2010). Gaps and ambiguous positions were removed from alignments prior to phylogenetic analysis. Mitochondrial (COI and 16S rDNA) and nuclear (ITS2 and H3) sequences were concatenated (Table 4) before phylogenetic analysis. Finally, the sequences of COI, 16S rDNA, ITS2 and H3 were concatenated (Table 4) for Maximum Likelihood (ML) and Bayesian Inference (BI).

**Table 4.** Concatenated sequences of COI+16S rDNA and ITS2+H3 for ML analysis (Figs 2, 3) and COI+16S rDNA+ITS2+H3 for Bayesian analysis (Fig. 4).

Concatenated sequence	COI haplotype	16S rDNA haplotype	Concatenated sequence	ITS2 common sequence	H3 common sequence	Concatenated sequence	COI haplotype	16S rDNA haplotype	ITS2 common sequence	H3 common sequence	Locality / population
*Monacha pantanellii* PAN											
COI16S 1	COI 33	16S 23	ITS2H3 1	ITS2 5	H3 6	CS 1	COI 33	16S 23	ITS2 5	H3 6	IT, Latium, Valle dell’Aniene [Ani]
COI16S 2	COI 34	16S 16	ITS2H3 2	ITS2 5	H3 1	CS 2	COI 34	16S 16	ITS2 5	H3 1	IT, Latium, Valle dell’Aniene
COI16S 3	COI 36	16S 25	ITS2H3 3	ITS2 14	H3 9	CS 3	COI 36	16S 25	ITS2 14	H3 9	IT, Latium, Valle dell’Aniene
			ITS2H3 4	ITS2 15	H3 14	CS 4	COI 36	16S 25	ITS2 15	H3 14	IT, Latium, Valle dell’Aniene
			ITS2H3 5	ITS2 7	H3 9	CS 5	COI 36	16S 25	ITS2 7	H3 9	IT, Latium, Valle dell’Aniene
COI16S 4	COI 35	16S 24	ITS2H3 6	ITS2 13	H3 15	CS 6	COI 35	16S 24	ITS2 13	H3 15	IT, Latium, Valle dell’Aniene
COI16S 5	COI 9	16S 7									IT, Latium, Ornaro Alto, Montenero Sabino [Sab]
COI16S 6	COI 11	16S 8				CS 7	COI 11	16S 8	ITS2 5	H3 1	IT, Latium, Ornaro Alto, Montenero Sabino
COI16S 7	COI 12	16S 8	ITS2H3 7	ITS2 6	H3 10	CS 8	COI 12	16S 8	ITS2 6	H3 10	IT, Latium, Ornaro Alto, Montenero Sabino
COI16S 8	COI 13	16S 9	ITS2H3 8	ITS2 6	H3 9	CS 9	COI 13	16S 9	ITS2 6	H3 9	IT, Latium, Ornaro Alto, Montenero Sabino
COI16S 9	COI 10	16S 8	ITS2H3 9	ITS2 4	H3 9	CS 10	COI 10	16S 8	ITS2 4	H3 9	IT, Latium, Ornaro Alto, Montenero Sabino
COI16S 10	COI 19	16S 8									IT, Abruzzi, Carsoli [Car]
COI16S 11	COI 31	16S 20	ITS2H3 10	ITS2 11	H3 9	CS 11	COI 31	16S 20	ITS2 11	H3 9	IT, Abruzzi, Carsoli
COI16S 12	COI 32	16S 21									IT, Abruzzi, Carsoli
COI16S 13	COI 8	16S 22									IT, Abruzzi, Carsoli
COI16S 14	COI 8	16S 19	ITS2H3 11	ITS2 5	H3 9	CS 12	COI 8	16S 19	ITS2 5	H3 9	IT, Abruzzi, Carsoli
COI16S 15	COI 25	16S 14				CS 13	COI 25	16S 14	ITS2 11	H3 9	IT, Latium, Lago del Turano (Castel di Tora, Rieti) [Tur2]
COI16S 16	COI 24	16S 13				CS 14	COI 24	16S 13	ITS2 5	H3 9	IT, Latium, Lago del Turano (Castel di Tora, Rieti)
COI16S 17	COI 26	16S 15				CS 15	COI 26	16S 15	ITS2 5	H3 1	IT, Latium, Lago del Turano (Castel di Tora, Rieti)
COI16S 18	COI 20	16S 8				CS 16	COI 20	16S 8	ITS2 5	H3 9	IT, Latium, Poggio San Lorenzo [Lor]
COI16S 19	COI 21	16S 10				CS 17	COI 21	16S 10	ITS2 5	H3 1	IT, Latium, Poggio San Lorenzo
						CS 18	COI 19	16S 8	ITS2 9	H3 6	IT, Latium, Poggio San Lorenzo
			ITS2H3 12	ITS2 9	H3 6	CS 19	COI 21	16S 10	ITS2 9	H3 6	IT, Latium, Poggio San Lorenzo
			ITS2H3 13	ITS2 9	H3 11	CS 20	COI 21	16S 10	ITS2 9	H3 11	IT, Latium, Poggio San Lorenzo
			ITS2H3 14	ITS2 3	H3 1	CS 21	COI 21	16S 10	ITS2 3	H3 1	IT, Latium, Poggio San Lorenzo
COI16S 20	COI 14	16S 8	ITS2H3 15	ITS2 7	H3 2	CS 22	COI 14	16S 8	ITS2 7	H3 2	IT, Latium, Ornaro Alto, Torricella in Sabina [Alt]
COI16S 21	COI 15	16S 10	ITS2H3 16	ITS2 3	H3 9	CS 23	COI 15	16S 10	ITS2 3	H3 9	IT, Latium, Ornaro Alto, Torricella in Sabina
COI16S 22	COI 16	16S 10	ITS2H3 17	ITS2 7	H3 11	CS 24	COI 16	16S 10	ITS2 7	H3 11	IT, Latium, Ornaro Alto, Torricella in Sabina
			ITS2H3 18	ITS2 8	H3 9	CS 25	COI 16	16S 10	ITS2 8	H3 9	IT, Latium, Ornaro Alto, Torricella in Sabina
COI16S 23	COI 18	16S 11	ITS2H3 19	ITS2 5	H3 12	CS 26	COI 18	16S 11	ITS2 5	H3 12	IT, Latium, Ornaro Alto, Torricella in Sabina
COI16S 24	COI 17	16S 10									IT, Latium, Ornaro Alto, Torricella in Sabina
COI16S 25	COI 30	16S 19				CS 27	COI 30	16S 19	ITS2 11	H3 9	IT, Latium, Valle del Turano (Turania, Rieti) [Tur1]
COI16S 26	COI 27	16S 16				CS 28	COI 27	16S 16	ITS2 11	H3 9	IT, Latium, Valle del Turano (Turania, Rieti)
			ITS2H3 20	ITS2 12	H3 10	CS 29	COI 27	16S 16	ITS2 12	H3 10	IT, Latium, Valle del Turano (Turania, Rieti)
COI16S 27	COI 28	16S 17	ITS2H3 21	ITS2 2	H3 1	CS 30	COI 28	16S 17	ITS2 2	H3 1	IT, Latium, Valle del Turano (Turania, Rieti)
			ITS2H3 22	ITS2 11	H3 1	CS 31	COI 22	16S 12	ITS2 11	H3 1	IT, Latium, Valle del Turano (Turania, Rieti)
COI16S 28	COI 29	16S 18				CS 32	COI 29	16S 18	ITS2 11	H3 1	IT, Latium, Valle del Turano (Turania, Rieti)
						CS 33	COI 24	16S 13	ITS2 2	H3 1	IT, Latium, Lago del Turano (Castel di Tora, Rieti) [Tur2]
COI16S 29	COI 22	16S 12	ITS2H3 23	ITS2 10	H3 13	CS 34	COI 22	16S 12	ITS2 10	H3 13	IT, Latium, Lago del Turano (Castel di Tora, Rieti)
COI16S 30	COI 23	16S 12									IT, Latium, Lago del Turano (Castel di Tora, Rieti)
COI16S 31	COI 8	16S 6									IT, Vallonina, Monti Reatini [Val]
COI16S 32	COI 7	16S 6									IT, Vallonina, Monti Reatini
COI16S 33	COI 4	16S 4	ITS2H3 24	ITS2 2	H3 2	CS 35	COI 4	16S 4	ITS2 2	H3 2	IT, Umbria, Monte Fionchi (680 m) [Fio2]
						CS 36	COI 6	16S 2	ITS2 2	H3 2	IT, Umbria, Monte Fionchi (680 m)
COI16S 34	COI 5	16S 5	ITS2H3 25	ITS2 3	H3 4	CS 37	COI 5	16S 5	ITS2 3	H3 4	IT, Umbria, Monte Fionchi (680 m)
COI16S 35	COI 6	16S 2	ITS2H3 26	ITS2 2	H3 5	CS 38	COI 6	16S 2	ITS2 2	H3 5	IT, Umbria, Monte Fionchi (680 m)
COI16S 36	COI 3	16S 3				CS 39	COI 3	16S 3	ITS2 2	H3 2	IT, Umbria, Monte Fionchi (680 m)
COI16S 37	COI 2	16S 1	ITS2H3 27	ITS2 1	H3 2	CS 40	COI 2	16S 1	ITS2 1	H3 2	IT, Umbria, Monte Fionchi (summit [Fio1]
COI16S 38	COI 1	16S 1									IT, Umbria, Monte Fionghi (summit)
*Monacha cantiana* CAN-1											
CAN-1	MG208916	MG208987	CAN-1	MH137974	MG209046	CS 41	MG208916	MG208987	MH137974	MG209046	IT, Latium, Valle dell’Aniene, Rome
	MG208915	MG208985		MH137973	MG209045	CS 42	MG208915	MG208985	MH137973	MG209045	IT, Latium, Valle dell’Aniene, Rome
	MG208917	MG208989		MH137975	MG209047	CS 43	MG208917	MG208989	MH137975	MG209047	IT, Latium, Valle dell’Aniene, Rome
	MG208905	MG208977				CS 44	MG208905	MG208977	MH137972	MG209039	IT, Latium, Gole del Velino
	MG208906	MG208979									IT, Latium, Gole del Velino
	MG208910	MG208978									IT, Latium, Gole del Velino
	MG208921	MG208990				CS 45	MG208921	MG208990	MH137976	MG209043	IT, Latium, Valle del Tronto
	MG208923	MG208994		MH137978	MG209048	CS 46	MG208923	MG208994	MH137978	MG209048	IT, Latium, Valle del Turano
	MG208884	MG208966				CS 47	MG208884	MG208966	MH137963	MG209031	UK, Barrow near Barnsley
	MG208899	MG208976		MH137971	MG209038	CS 48	MG208899	MG208976	MH137971	MG209038	UK, Rotherham
	MG208893	MG208960									UK, Rotherham
	MG208898	MG208975		MH137969	MG209037	CS 49	MG208898	MG208975	MH137969	MG209037	UK, Sheffield
	MG208904	MG208971									UK, Sheffield
	MG208891	MG208972									UK, Cambridge
*Monacha cantiana* CAN-2											
CAN-2	MG208925	MG208996	CAN-2	MK067000	MG209050						IT, Venetum, Sorga
	MG208926	MG209001									IT, Venetum, Sorga
	MG208928	MG208998									IT, Venetum, Sorga
	MG208932	MG209003		MH137981	MG209052	CS 50	MG208932	MG209003	MH137981	MG209052	IT, Lombardy, Rezzato
*Monacha cantiana* s. l. CAN-3 (*Monacha* sp.)											
CAN-3	MG208936	MG209009	CAN-3	MH137983	MG209055	CS 51	MG208936	MG209009	MH137983	MG209055	AU, Breitenlee
	MG208938	MG209008									AU, Breitenlee
	MG208933	MG209007		MH137982	MG209054	CS 52	MG208933	MG209007	MH137982	MG209054	IT, Emilia Romagna, Fiume Setta
	MG208934	MG209005									IT, Emilia Romagna, Fiume Setta
	MG208935	MG209006									IT, Emilia Romagna, Fiume Setta
*Monacha cantiana* s. l. CAN-4 (*Monacha cemenelea)*											
CAN-4	MG208939	MG209011	CAN-4	MH137984	MG209058	CS 53	MG208939	MG209011	MH137984	MG209058	FR, Alpes-Maritimes, Sainte Thecle
	MG208940	MG209012									FR, Alpes-Maritimes, Sainte Thecle
	MG208941	MG209013									FR, Alpes-Maritimes, Sainte Thecle
				MK067003	MG209059						FR, Alpes-Maritimes, Sainte Thecle
*Monacha cantiana* s. l. CAN-5 (*Monacha* sp.)											
CAN-5	MK066929	MK066947	CAN-5								IT, Tuscany, Foce di Pianza
	MK066933	MK066951									IT, Tuscany, Foce di Pianza
						CS 54	MK066931	MK066949	MK066982	MK066967	IT, Tuscany, Foce di Pianza
				MK066981	MK066966	CS 55	MK066930	MK066948	MK066981	MK066966	IT, Tuscany, Foce di Pianza
				MK066983	MK066968	CS 56	MK066932	MK066950	MK066983	MK066968	IT, Tuscany, Foce di Pianza
	MK066935	MK066954		MK066987	MK066972	CS 57	MK066935	MK066954	MK066987	MK066972	IT, Tuscany, Campo Cecina
	MK066937	MK066956		MK066989	MK066974	CS 58	MK066937	MK066956	MK066989	MK066974	IT, Tuscany, Campo Cecina
	MK066934	MK066952		MK066985	MK066970	CS 59	MK066934	MK066952	MK066985	MK066970	IT, Tuscany, Campo Cecina
	MK066936	MK066955		MK066988	MK066973	CS 60	MK066936	MK066955	MK066988	MK066973	IT, Tuscany, Campo Cecina
	MK066938	MK066957		MK066991	MK066976	CS 61	MK066938	MK066957	MK066991	MK066976	IT, Piastra
	MK066939	MK066958									IT, Piastra
	MK066941	MK066959									IT, Piastra
*Monacha cantiana* s. l. CAN-6 (*Monacha* sp.)											
CAN-6	MK066942	MK066960	CAN-6								IT, Tuscany, Campagrina
	MK066943	MK066961									IT, Tuscany, Campagrina
	MK066944	MK066962				CS 62	MK066944	MK066962	MK066997	MK066978	IT, Tuscany, Campagrina
	MK066945	MK066963									IT, Tuscany, Campagrina
				MK066999	MK066980	CS 63	MK066946	MK066964	MK066999	MK066980	IT, Tuscany, Campagrina
*Monacha parumcincta* PAR											
PAR	MG208946	MG209019	PAR								IT, Basilicata, Moliterno to Fontana d’Eboli
	MG208947	MG209016									IT, Basilicata, Moliterno to Fontana d’Eboli
				MK067005	MG209061	CS 64	MG208944	MG209017	MK067005	MG209061	IT, Basilicata, Moliterno to Fontana d’Eboli
				MH137992	MG209064						IT, Basilicata, Moliterno to Fontana d’Eboli
	MG208949	MG209020		MH137987	MG209067	CS 65	MG208949	MG209020	MH137987	MG209067	IT, Tuscany, Nievole
	MG208953	MG209021									IT, Tuscany, Nievole & Arezzo
	MG208950	MG209028									IT, Tuscany, Arezzo
				MH137989	MG209068						IT, Tuscany, Arezzo
	MG208956	MG209025		MH137990	MG209070	CS 66	MG208956	MG209025	MH137990	MG209070	IT, Tuscany, Arezzo
	MG209959	MG209030		MH137986	MG209062	CS 67	MG208959	MG209030	MJ137986	MG209062	IT, Tuscany, Arezzo & La Casella
*Monacha cartusiana* CAR											
CAR	MH203998	MH204081	CAR								DE, Lower Saxony, Hannover, Sehnde
				MH137993	MG209072	CS 68	KM247376	KM247391	MH137993	MG209072	HU, Kis-Balaton

Estimates of evolutionary divergence between the sequences of COI obtained in this study and other sequences from GenBank were conducted with MEGA7 using the Kimura two-parameter model (K2P) (Kimura 1980). The analysis involved 83 nucleotide sequences. All positions containing gaps and missing data were eliminated. There was a total of 615 positions in the final dataset.

Maximum Likelihood (ML) analyses were then performed with MEGA7. *Monacha cartusiana* and *Monacha parumcincta* were added as outgroup species in each analysis. For ML analysis of concatenated sequences, the following best nucleotide substitution models were specified according to the Bayesian Information Criterion (BIC): HKY+G+I (Hasegawa et al. 1985, Kumar et al. 2016) for COI and 16S rDNA concatenated sequences of 878 positions (592 COI + 286 16S rDNA), T92+G+I (Tamura 1992, Kumar et al. 2016) for ITS2+H3 concatenated sequences of 780 positions (501 ITS2 + 279 H3), and T92+G+I for COI+16S rDNA+ITS2+H3 concatenated sequences with a total length of 1658 positions (592 COI + 286 16S rDNA + 501 ITS2 + 279 H3). Bayesian analysis was conducted with MrBayes 3.1.2 (Ronquist and Huelsenbeck 2003) using the evolution model already used for ML calculation. Four Monte Carlo Markov chains were run for one million generations, sampling every 100 generations (the first 250,000 trees were discarded as ‘burn-in’). This gave us a 50% majority rule consensus tree. In parallel, Maximum Likelihood (ML) analysis was performed with MEGA7 (Kumar et al. 2016) and calculated bootstrap values were mapped on the 50% majority rule consensus Bayesian tree.

### Morphological study

One hundred and thirty-four specimens representing *M. pantanellii*, *M. cantiana* s. l., *M. parumcincta* and *M. cartusiana* were considered to investigate shell variability between these four species (including six molecular lineages of *M. cantiana* s. l.) (see Table 1 and Pieńkowska et al. 2018a, 2019b); the 43 specimens of nine populations of *M. pantanellii* (Fio1, Val, Sab, Alt, Lor, Tur2, Tur1, Car and Ani, see Table 1) were also considered to investigate shell variability between specimens of these populations. Shell variability was analysed randomly choosing five adult specimens from each population, when possible. Twelve shell variables were measured to the nearest 0.1 mm using Adobe Photoshop 7.0.1 on digital images of apertural and umbilical standard views taken with a Canon EF 100 mm 1:2.8 L IS USM macro lens mounted on a Canon F6 camera: **AH** aperture height, **AW** aperture width, **LWfW** last whorl final width, **LWmW** last whorl medial width, **LWaH** height of adapical sector of last whorl, **LWmH** height of medial sector of last whorl, **PWH** penultimate whorl height, **PWfW** penultimate whorl final width, **PWmW** penultimate whorl medial width, **SD** shell diameter, **SH** shell height, and **UD** umbilicus diameter (Pieńkowska et al. 2018a: fig. 1).

One hundred and thirty-five specimens of *M. pantanellii*, *M. cantiana* s. l. (with its six molecular lineages), *M. parumcincta* and *M. cartusiana* were analysed to examine anatomical variability between species; the 50 specimens of ten populations of *M. pantanellii* were also considered to investigate genital variability between populations of this species (see Table 1 and Pieńkowska et al. 2018a, 2019b). Snail bodies were dissected under the light microscope (Wild M5A or Zeiss SteREO Lumar V12). Anatomical details were drawn using a Wild camera lucida. Acronyms: **BC** bursa copulatrix, **BW** body wall, **DBC** duct of bursa copulatrix, **DG** digitiform glands (also known as mucous glands), **E** epiphallus (from base of flagellum to beginning of penial sheath), **F** flagellum, **FO** free oviduct, **GA** genital atrium, **GAR** genital atrium retractor, **OSD** ovispermiduct, **P** penis, **PP** penial papilla (also known as glans), **V** vagina, **VA** vaginal appendix (also known as appendicula), **VAS** vaginal appendix basal sac, **VS** vaginal sac (only present in *M. cartusiana*; see Pieńkowska et al. 2015: figs 11, 12), **VD** vas deferens. Seven anatomical variables (DBC, E, F, P, V, VS, VA) were measured under a light microscope (0.01 mm) using callipers (see: Pieńkowska et al. 2018a: fig. 2).

Detailed methods of multivariate ordination by Principal Component Analysis (PCA) and Redundancy Analysis (RDA), performed on the original shell and genitalia matrices as well as on the shape-related Z-matrices, are described in a previous paper (Pieńkowska et al. 2018a).

Differences between species for each shell and genital character were assessed through box-plots and descriptive statistics. Overall significance of differences was obtained using the Kruskal-Wallis test; when the test proved significant, multiple comparisons between pairs of species were performed using Dunn’s test. In order to control the false discovery rate (FDR), the Benjamini-Hochberg correction was used to adjust P-values for multiple comparisons. The dunn.test function with the altp = TRUE option and α = 0.01 in the dunn.test R package were used for analysis (Dinno 2017).

## Results

### Molecular study

**Figure 2.**
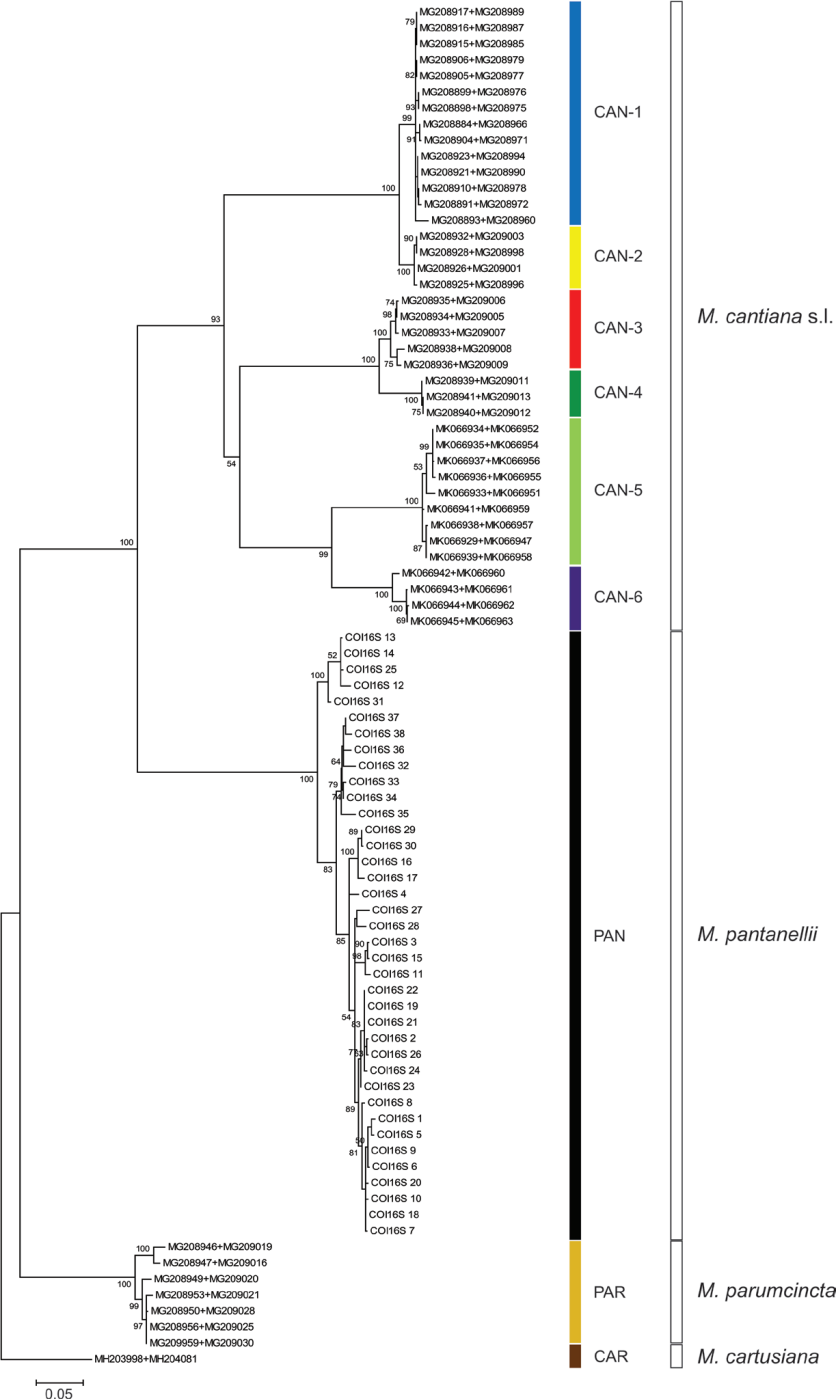
Maximum Likelihood (ML) tree of concatenated COI and 16S rDNA haplotypes of *Monacha pantanellii* (see Table 4). New COI and 16S rDNA sequences of *M. pantanellii* (Table 1) were compared with COI and 16S rDNA sequences of *M. cantiana* s. l. and *M. parumcincta* obtained from GenBank (Tables 2, 4). Numbers next to branches indicate bootstrap support above 50% calculated on 1000 replicates (Felsenstein 1985). The tree was rooted with *M. cartusiana* concatenated sequences obtained from GenBank (Table 2).

**Figure 3.**
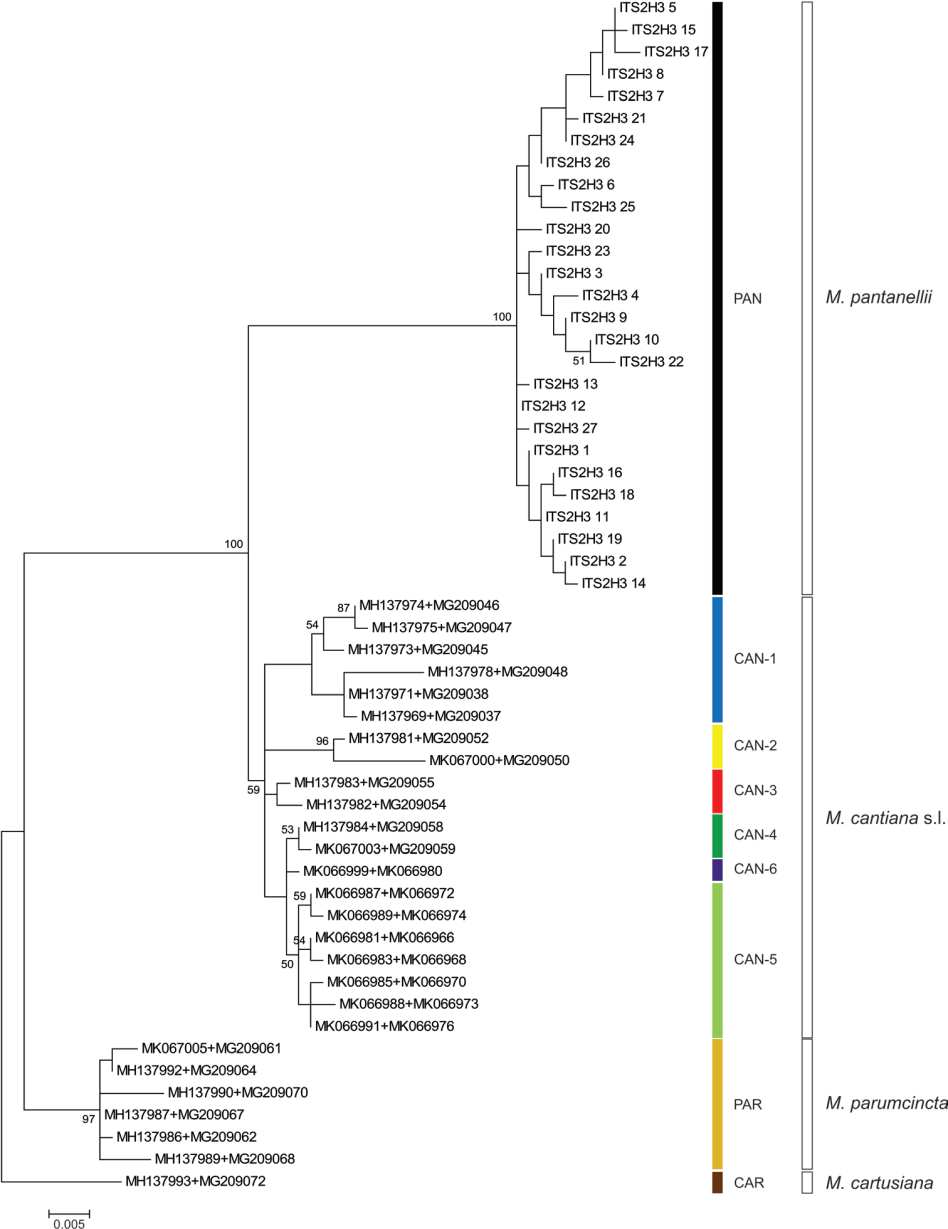
Maximum Likelihood (ML) tree of concatenated ITS2 and H3 common sequences of *Monacha pantanellii* (see Table 4). New ITS2 and H3 sequences of *M. pantanellii* (Table 1) were compared with ITS2 and H3 sequences of *M. cantiana* s. l. and *M. parumcincta* obtained from GenBank (Tables 2, 4). Numbers next to branches indicate bootstrap support above 50% calculated on 1000 replicates (Felsenstein 1985). The tree was rooted with *M. cartusiana* concatenated sequences obtained from GenBank (Table 2).

**Figure 4.**
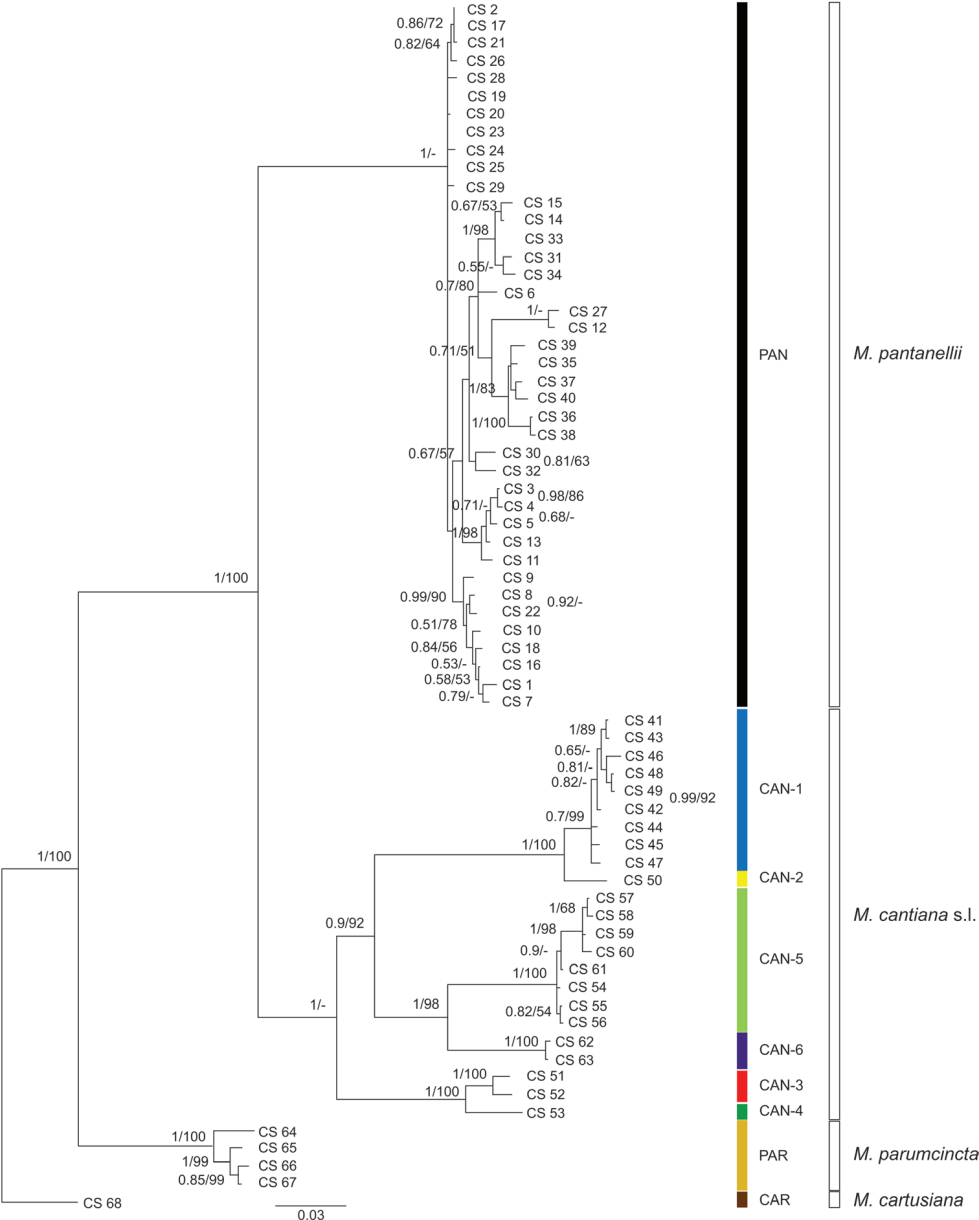
Bayesian 50% majority-rule consensus tree of the concatenated data set of COI and 16S rDNA haplotypes, and ITS2 and H3 common sequences (see Table 4). Sequences of *M. pantanellii* were compared with appropriate sequences of *M. cantiana* s. l. and *M. parumcincta* obtained from GenBank (Tables 2, 4). Posterior probabilities (left) and bootstrap support above 50% from ML analysis (right) are indicated next to the branches. Bootstrap analysis was run with 1000 replicates (Felsenstein 1985). The tree was rooted with *M. cartusiana* concatenated sequences obtained from GenBank (Table 2).

DNA sequencing resulted in 53 and 57 sequences of mitochondrial COI and 16S rDNA as well as 43 and 57 sequences of nuclear ITS2 and H3 gene fragments, respectively. They were all deposited in GenBank as MT380011–MT380063 (COI), MT376031–MT376087 (16S rDNA), MT376088–MT376130 (ITS2) and MT385776–MT385832 (H3) (Table 1). Thirty-six COI (COI 1–COI 36) and 25 16S rDNA (16S 1–16S 25) haplotypes, as well as 15 ITS2 (ITS2 1–ITS2 15) and 15 H3 (H3 1–H3 15) common sequences were recognised among them (Table 1). They were used for phylogenetic analysis with appropriate sequences representing *M. parumcincta* (PAR) and *M. cartusiana* (CAR), as well as six molecular lineages of *M. cantiana* s. l. (CAN-1–CAN-6) obtained from GenBank (Table 2). ML trees for concatenated sequences of mitochondrial COI and 16S rDNA (Fig. 2, Table 4) and of nuclear ITS2 and H3 (Fig. 3, Table 4) gene fragments, as well as the Bayesian Inference (BI) phylogenetic tree of concatenated sequences of COI+16S rDNA+ITS2+H3 gene fragments (Fig. 4, Table 4) clustered the concatenated sequences in one clade (PAN) separated from all other clades hitherto recognised for *M. cantiana* s. l. (CAN-1, CAN-2, CAN-3, CAN-4, CAN-5, CAN-6), *M. parumcincta* (PAR) and *M. cartusiana* (CAR) populations (Pieńkowska et al. 2018a, 2018b, 2019b).

K2P genetic distances between COI haplotypes are summarised in Table 5. Differences in COI haplotypes of *M. pantanellii* are rather small (up to 4.5%). Three varied somewhat more (COI 8 from populations from Vallonina [Val] and Carsoli [Car], COI 30 from Valle del Turano [Tur1] and COI 32 from Carsoli [Car]), bringing the mean for all populations to 0.2–6.7%. It was not possible to differentiate one population from the others. It is noteworthy that haplotypes of *M. pantanellii* are very different (15.5–22.0%) from the others representing *M. cantiana* s. l. (i.e., *M. cantiana* CAN-1–CAN-3, *M. cemenelea* (Risso, 1826) CAN-4, and *M.* sp. CAN-5–CAN-6) as well as from *M. parumcincta* (18.1–21.4%) and *M. cartusiana* (16.6–18.3%) (Pieńkowska et al. 2018a, 2019b).

**Table 5.** Ranges of K2P genetic distances between analysed COI sequences.

Comparison	COI (%)
Within *M. pantanellii* PAN	0.2–6.7
Between *M. pantanellii* PAN and *M. cantiana* CAN-1	17.2–21.2
Between *M. pantanellii* PAN and *M. cantiana* CAN-2	19.1–22.0
Between *M. pantanellii* PAN and *M. cantiana* s. l. CAN-3 (*M.* sp.)	16.8–18.9
Between *M. pantanellii* PAN and *M. cantiana* s. l. CAN-4 (*M. cemenelea*)	15.5–17.4
Between *M. pantanellii* PAN and *M. cantiana* s. l. CAN-5 (*M.* sp.)	17.1–19.9
Between *M. pantanellii* PAN and *M. cantiana* s. l. CAN-6 (*M.* sp.)	15.5–18.6
Between *M. pantanellii* PAN and *M. parumcincta*PAR	18.1–21.4
Between *M. pantanellii* PAN and *M. cartusiana*CAR	16.6–18.3

### Morphological study: shell

*Monacha pantanellii* has a globose to sub-globose shell, variable in size, colour, and presence of paler subsutural and peripheral bands, with roundish to oval slightly descending aperture, a brownish peristome and a very small to small umbilicus (Figs 5–31).

**Figures 5–14.**
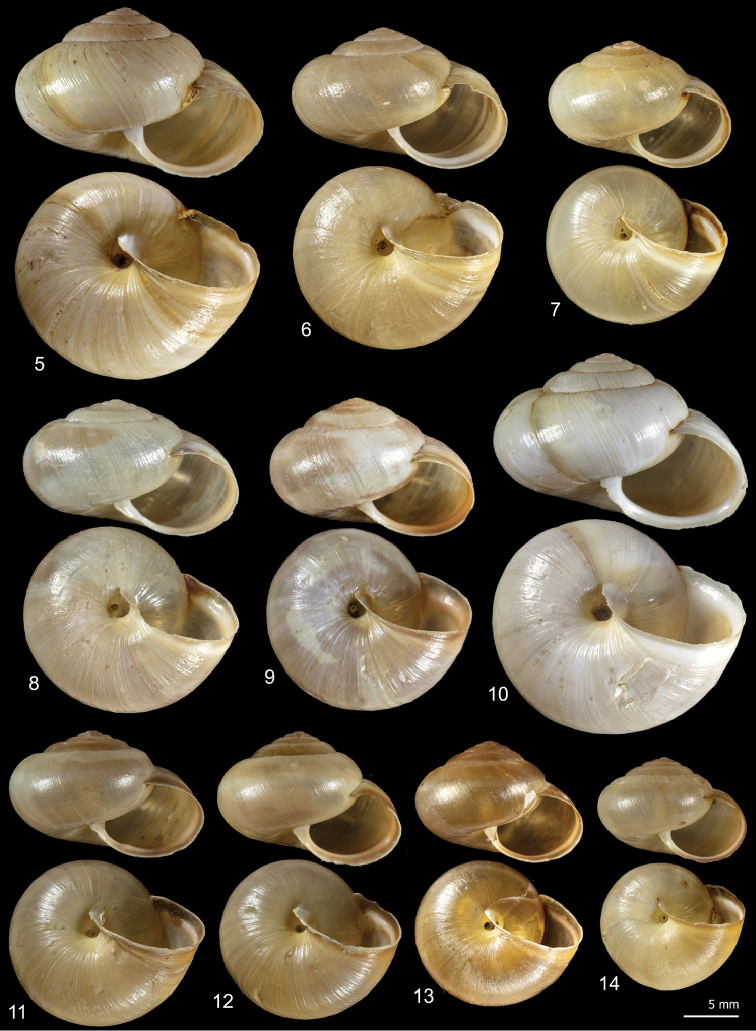
Shell variability in *Monacha pantanellii* from Monte Fionchi, summit [Fio1] (FGC 8140) (**5, 6**), Monte Fionchi, Torrecola [Fio2] (FGC 38944) (**7**), road to Montenero Sabino [Sab] (FGC 41552) (**8–10**) and Turania [Tur1] (FGC 42971) (**11–14**).

**Figures 15–22.**
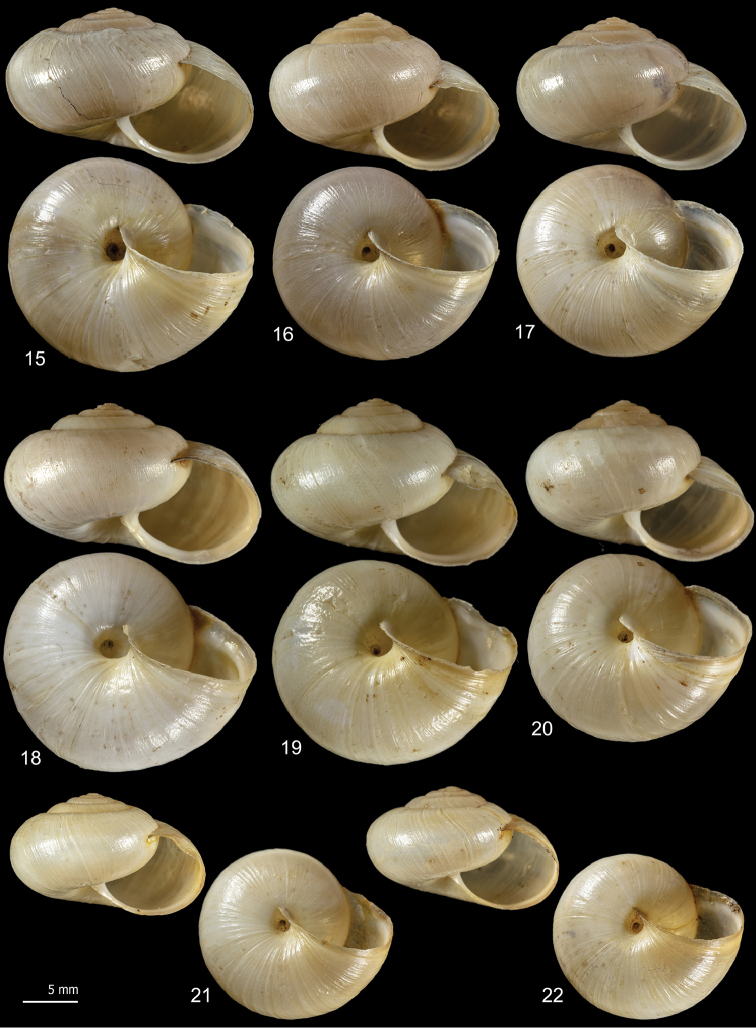
Shell variability in *Monacha pantanellii* from Lago del Turano [Tur2] (FGC 41654) (**15–17**), Via Salaria, Ornaro Alto [Alt] (FGC 41553) (**18–20**) and Vallonina [Val] (FGC 25345) (**21, 22**).

**Figures 23–31.**
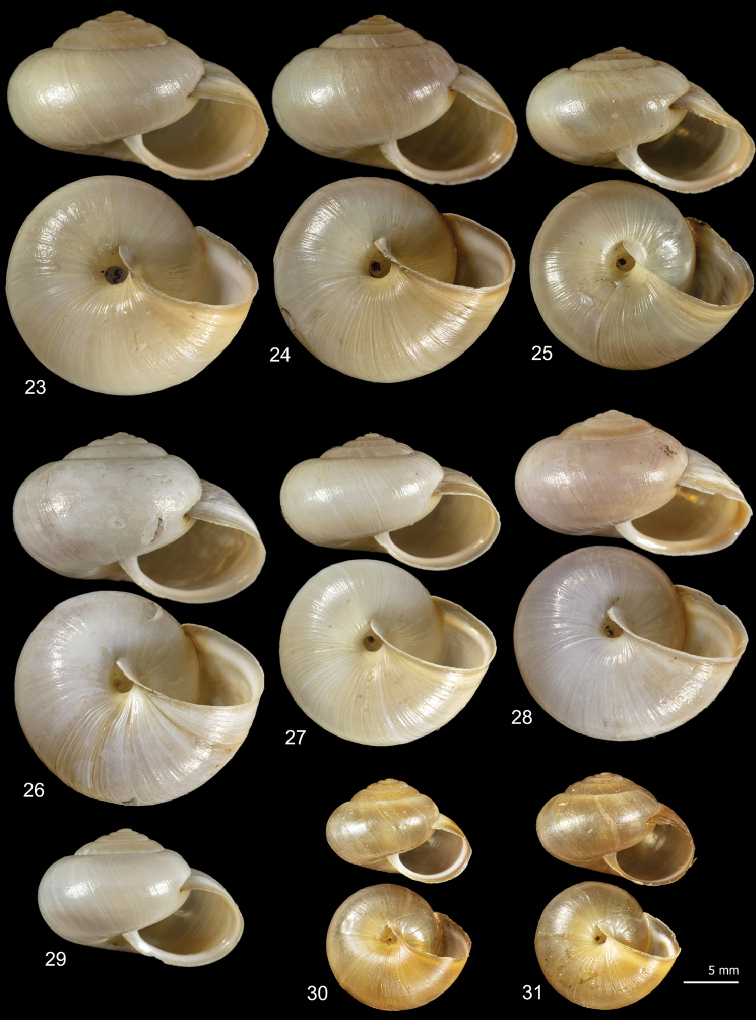
Shell variability in *Monacha pantanellii* from Via Salaria, Poggio San Lorenzo [Lor] (FGC 41551) (**23–25**), Valle dell’Aniene, Roccagiovine [Ani] (FGC 42974) (**26–29**) and Carsoli [Car] (FGC 41651(**30, 31**).

**Figures 32, 33.**
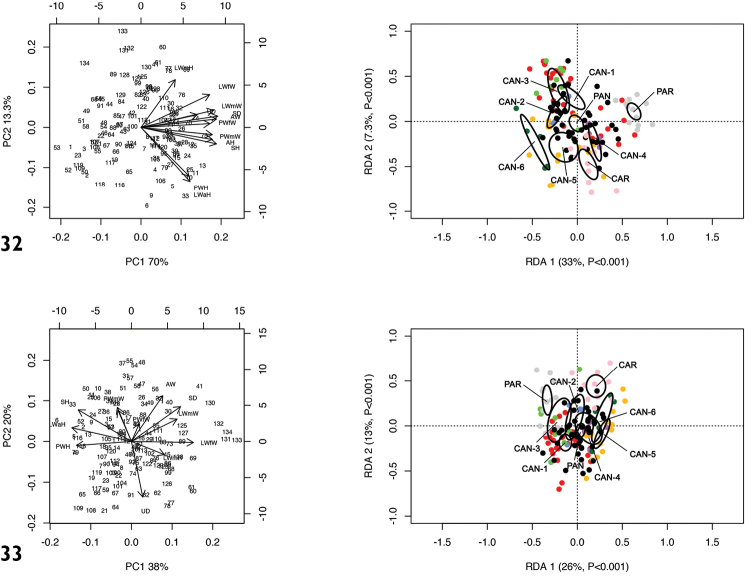
Principal component analysis (PCA) and Redundancy analysis (RDA) with species or molecular lineage constraint applied to the original shell matrix (**32**) and shape-related Z-matrix (**33**).

RDA with species or molecular lineage constraint on the shape and size matrix (Fig. 32) showed that RDA 1 (33%, P < 0.001) separated all the species or molecular lineages from PAR. The preliminary classic PCA revealed size as the first major source of morphological variation, since PC1 (70%) was a positive combination of all variables. On the contrary, RDA 2 (7.3%, P < 0.001) slightly separated CAN-1, CAN-2 and CAN-3 from CAN-4, CAN-5, CAN-6 and PAN with PAR in intermediate position. In this regard, PC2 (13%) mostly accounted for contrast between LWmH vs. LWaH and PWH.

RDA on the shape (Z) matrix (Fig. 33) showed a hazier separation of species or molecular lineages, confirming that size is a major source of morphological variation, although both RDA axes proved to be significant. In particular, RDA 1 separated CAR, CAN-5, CAN-6 from PAR, CAN-1 and CAN-3, with the other groups in a more or less intermediate position. Conversely, RDA 2 separated PAR and CAR from all the other species or molecular lineages. Shape-related PCA indicated that SH, LWaH and PWH vs. LWfW were the principal shape determinants on PC1 and PWmW, AH and AD vs. UD on PC2.

**Figure 34.**
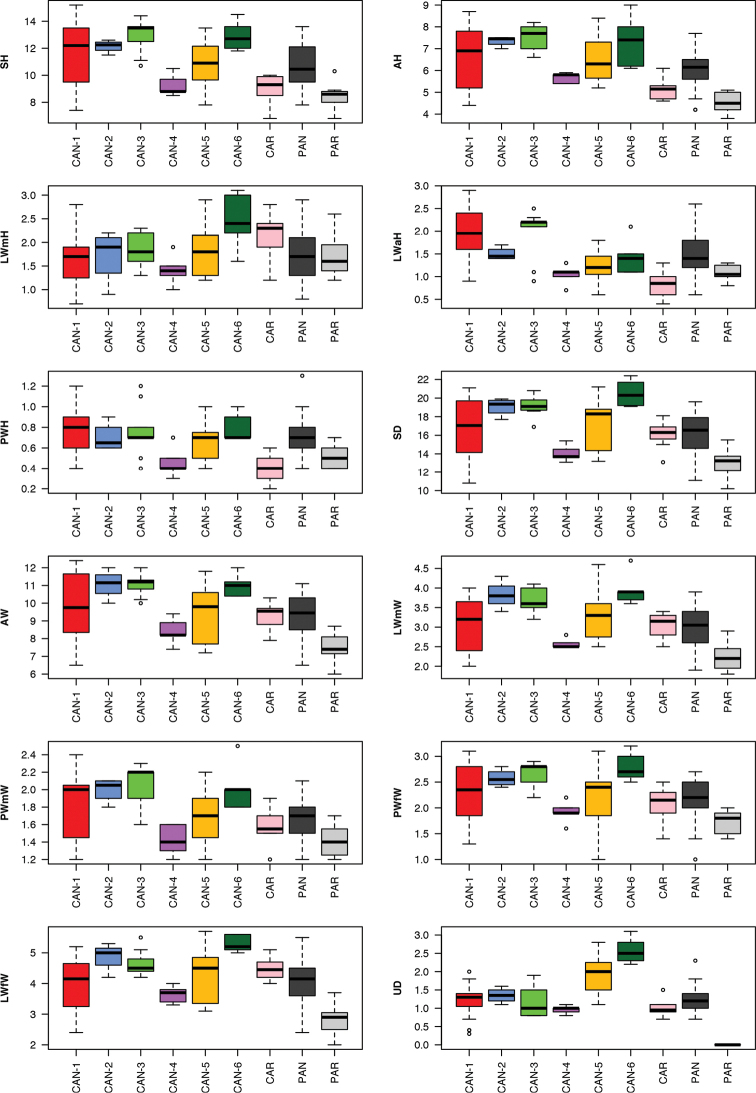
Box plots for shell characters of the nine *Monacha* species or molecular lineages investigated. The lower and upper limits of the rectangular boxes indicate the 25^th^ to 75^th^ percentile range, and the horizontal line within the boxes is the median (50^th^ percentile).

Box plots (Fig. 34) proved that the shell characters only have discriminating value in distinguishing *Monacha pantanellii* from other species or molecular lineages in a few cases. In fact, according to Dunn’s test with Benjamini-Hochberg adjustment (α = 0.01), no character significantly distinguished PAN from CAN-1, CAN-2 and CAN-4, only one distinguished it from CAN-5 (UD), only two from CAR (LWah, PWH), four from CAN-6 (SD, LWmW, LWfW, UD), six from CAN-3 (SH, AH, SD, AD, LWmW, PWmW) and eight from PAR (SH, AH, SD, AD, LWmW, PWfW, LWfW, UD) (Table 6).

**Table 6.** Results of Dunn’s test with Benjamini-Hochberg correction (α = 0.01) for shell and genital characters (in bold P ≤ 0.01).

Pairs	SH	AH	LWmH	LWaH	PWH	SD	
PAN vs. CAN-1	0.0956	0.1431	0.3784	0.0134	0.1993	0.2703	
PAN vs. CAN-2	0.2257	0.0763	0.9541	0.8128	0.9275	0.0517	
PAN vs. CAN-3	**0.0075**	**0.0039**	0.7552	0.1309	0.6223	**0.0063**	
PAN vs. CAN-4	0.1428	0.4689	0.3232	0.0750	0.0467	0.1496	
PAN vs. CAN-5	0.8439	0.4087	0.8724	0.1396	0.8163	0.3364	
PAN vs. CAN-6	0.0514	0.0895	0.1007	0.8442	0.3559	**0.0039**	
PAN vs. CAR	0.0468	0.0330	0.1163	**0.0009**	**0.0026**	0.7972	
PAN vs. PAR	**0.0022**	**0.0003**	0.7724	0.0110	0.0227	**0.0044**	
**Pairs**	**AW**	**LWmW**	**PWmW**	**PWfW**	**LWfW**	**UD**	
PAN vs. CAN-1	0.1792	0.5046	0.0468	0.4863	0.8655	0.9405	
PAN vs. CAN-2	0.0488	0.0189	0.0434	0.1789	0.0826	0.5901	
PAN vs. CAN-3	**0.0054**	**0.0046**	**0.0085**	0.0265	0.0711	0.5962	
PAN vs. CAN-4	0.3094	0.1947	0.1515	0.1979	0.3344	0.1765	
PAN vs. CAN-5	0.8931	0.2051	0.7961	0.8167	0.3478	**0.0015**	
PAN vs. CAN-6	0.0330	**0.0043**	0.0434	0.0249	**0.0030**	**0.0029**	
PAN vs. CAR	1.0000	0.9480	0.4609	0.4984	0.1652	0.1370	
PAN vs. PAR	**0.0046**	**0.0028**	0.0365	**0.0054**	**0.0008**	**0.0000**	
**Pairs**	**DBC**	**V**	**F**	**E**	**P**	**VA**	**VS**
PAN vs. CAN-1	0.3802	0.0992	**0.0000**	**0.0072**	**0.0001**	**0.0000**	1.0000
PAN vs. CAN-2	0.0808	0.1870	**0.0001**	**0.0003**	0.5535	**0.0000**	1.0000
PAN vs. CAN-3	0.9561	0.4778	**0.0000**	**0.0057**	0.5350	**0.0000**	1.0000
PAN vs. CAN-4	0.3528	0.9287	0.0708	0.9913	**0.0001**	**0.0013**	1.0000
PAN vs. CAN-5	0.0813	0.1862	0.6815	**0.0002**	**0.0006**	**0.0001**	1.0000
PAN vs. CAN-6	0.1163	0.3350	0.7574	0.0328	0.0101	**0.0001**	1.0000
PAN vs. CAR	**0.0009**	0.2609	**0.0000**	0.0122	**0.0000**	0.6581	**0.0000**
PAN vs. PAR	0.0430	**0.0000**	**0.0000**	0.1266	**0.0000**	0.5918	1.0000

**Figures 35, 36.**
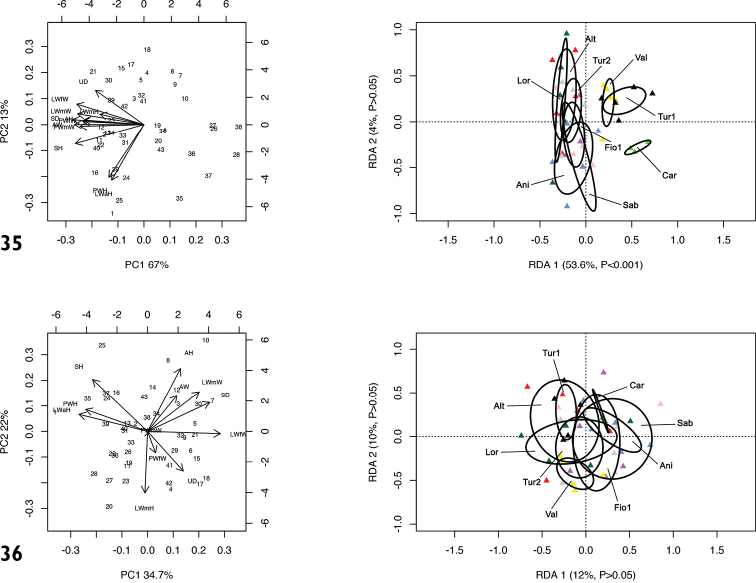
Principal component analysis (PCA) and Redundancy analysis (RDA) with population constraint applied to the original shell matrix (**35**) and shape-related Z-matrix (**36**) of specimens of *Monacha pantanellii*.

RDA with population constraint on the shape and size matrix (Fig. 35) showed that RDA 1 (53.6%, P < 0.001) separated them into two groups, the first, including populations from Via Salaria, Ornaro Alto [Alt], Valle dell’Aniene, Roccagiovine [Ani], Monte Fionchi, summit [Fio1], Via Salaria, Poggio San Lorenzo [Lor], Montero Sabino [Sab] and Lago del Turano [Tur2] was separate from the second consisting of populations from Carsoli [Car], Turania [Tur1] and Vallonina [Val]. On the contrary, RDA 2 (4.0%, P > 0.05) showed no significant separation of populations. The preliminary classic PCA revealed size as the first major source of morphological variation, since PC1 (67.0%) was a negative combination of all variables.

RDA on the shape (Z) matrix (Fig. 36) showed no significant separation between populations, again confirming that size is a major source of morphological variation. Shape-related PCA indicated that LWaH and PWH vs. LWfW were the two principal shape determinants on PC1 and AH vs. LWmH on PC2.

### Morphological study: anatomy

*Monacha pantanellii* has distal genitalia very similar to those of the *Monacha cantiana* group. The most remarkable features are the usually short vaginal appendix with mid or proximal vaginal insertion, the long flagellum and the penial papilla with thick external wall bordering a central duct without strips joining it to the external wall and with a lumen filled by many variably sized pleats (Figs 37–63).

**Figures 37–40.**
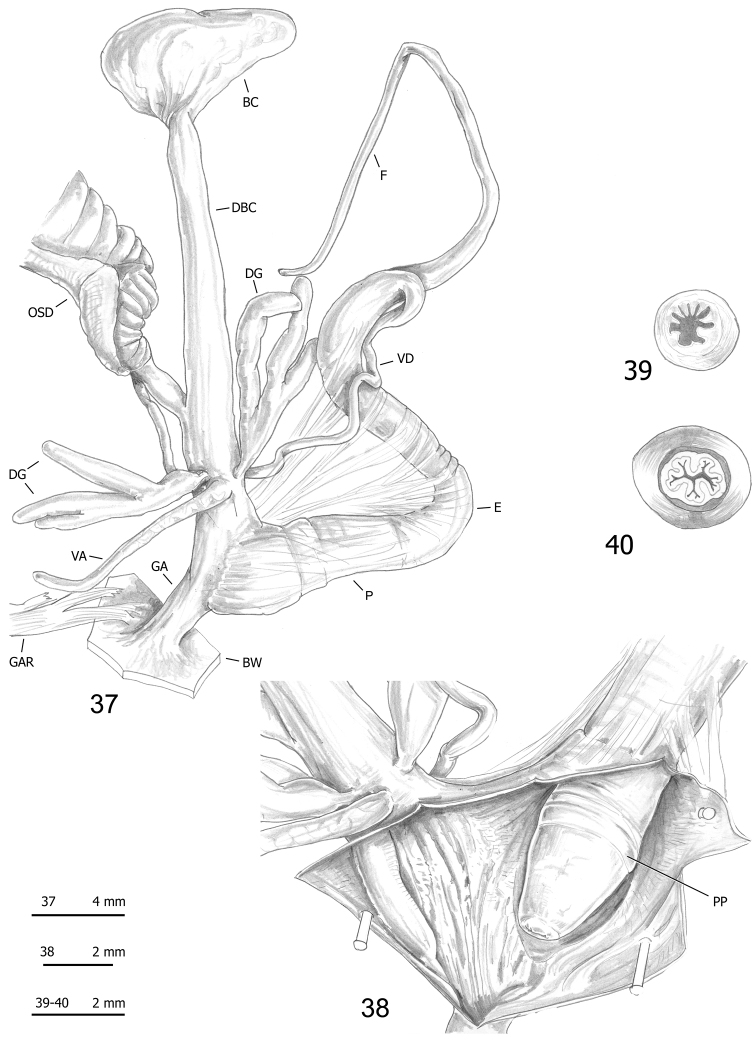
Genitalia (proximal parts excluded) (**37**), internal structure of distal genitalia (**38**), transverse sections of medial epiphallus (**39**) and apical penial papilla (**40**) of *Monacha pantanellii* from Monte Fionchi summit [Fio1] (FGC 8140).

**Figures 41–44.**
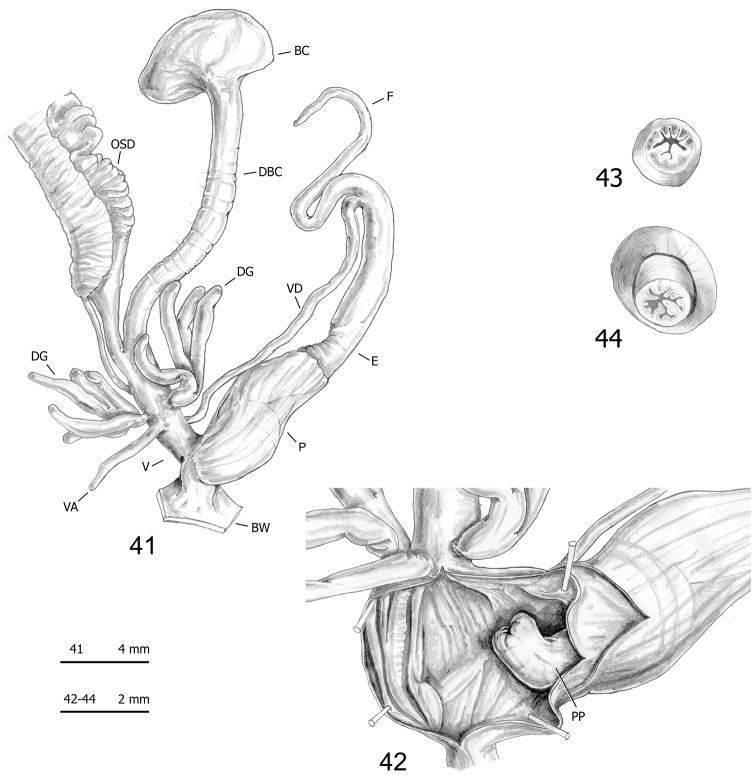
Genitalia (proximal parts excluded) (**41**), internal structure of distal genitalia (**42**), transverse sections of medial epiphallus (**43**) and apical penial papilla (**44**) of *Monacha pantanellii* from Monte Fionchi, Torrecola [Fio2] (FGC 38944).

**Figures 45–48.**
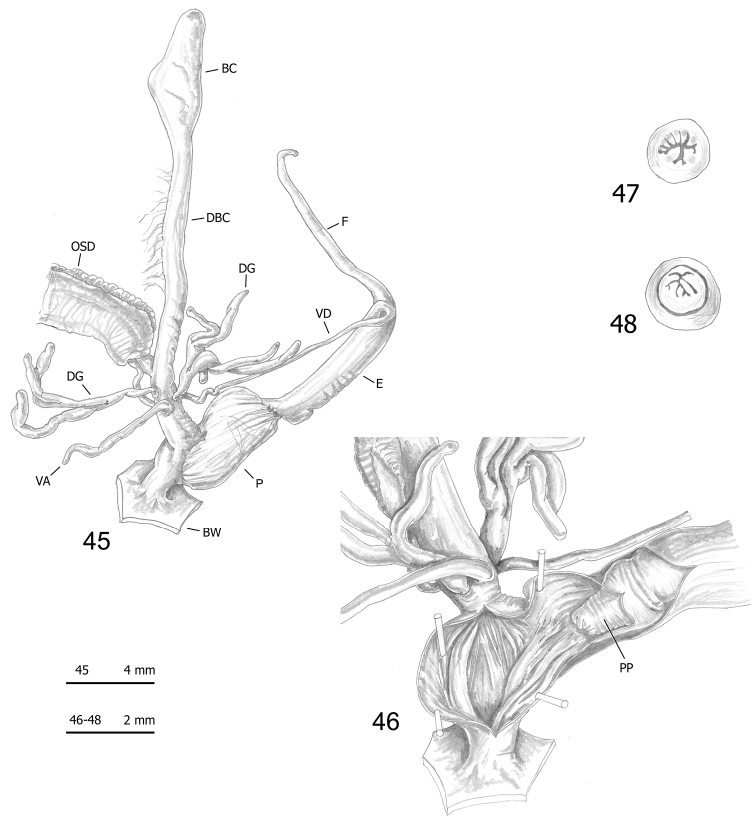
Genitalia (proximal parts excluded) (**45**), internal structure of distal genitalia (**46**), transverse sections of medial epiphallus (**47**) and apical penial papilla (**48**) of *Monacha pantanellii* from Vallonina [Val] (FGC 25345).

**Figures 49–52.**
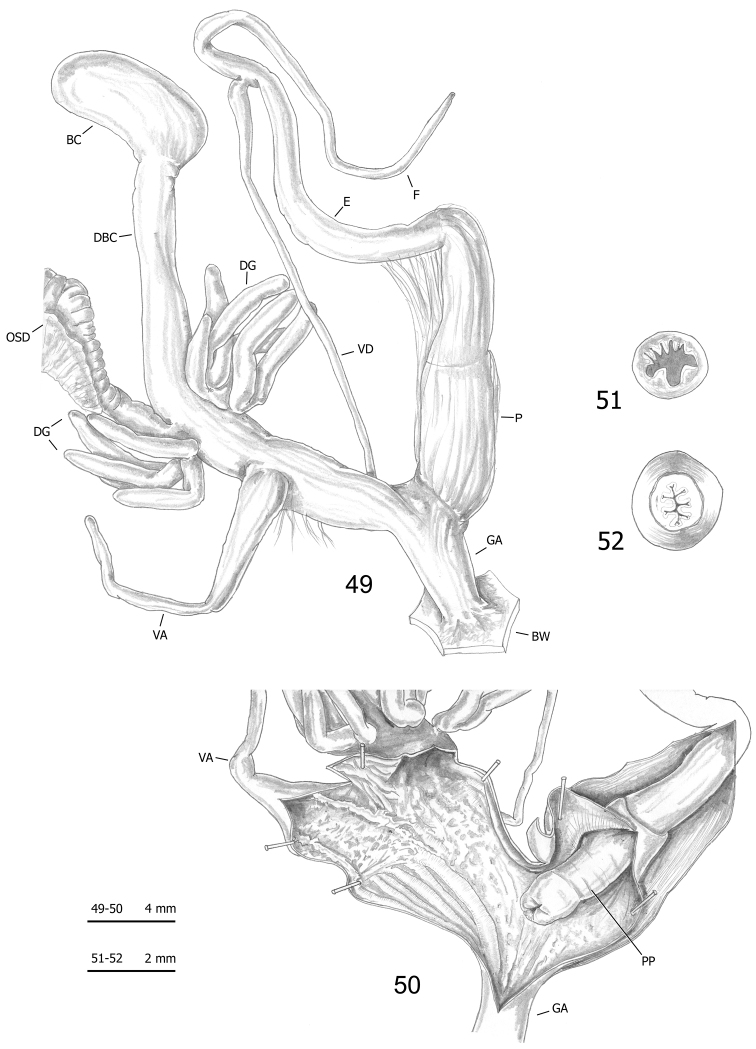
Genitalia (proximal parts excluded) (**49**), internal structure of distal genitalia (**50**), transverse sections of medial epiphallus (**51**) and apical penial papilla (**52**) of *Monacha pantanellii* from Valle dell’Aniene, Roccagiovine [Ani] (FGC 42974).

**Figures 53–56.**
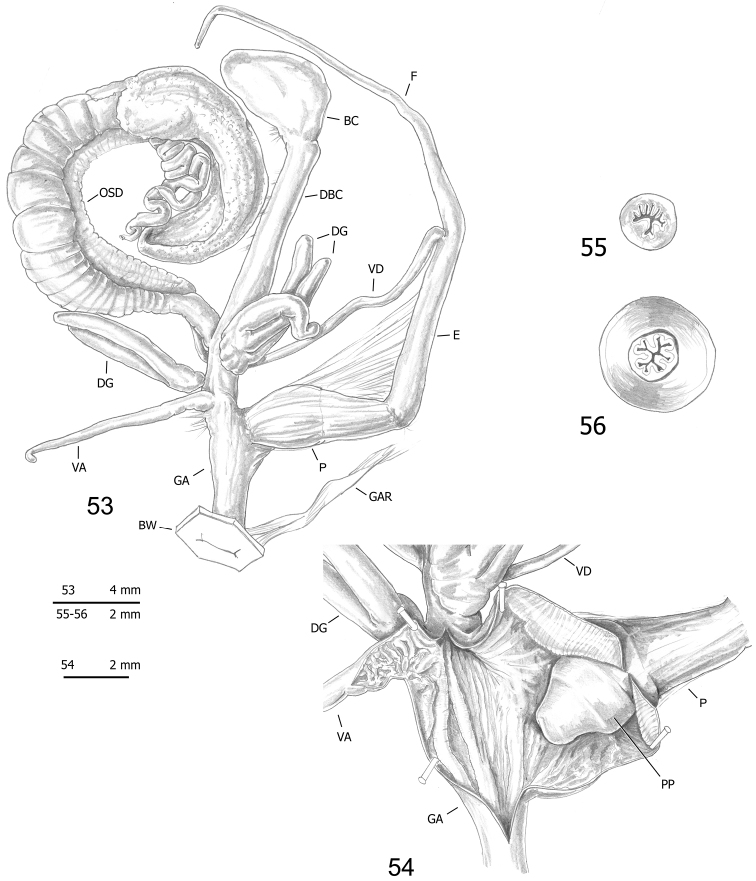
Genitalia (proximal parts excluded) (**53**), internal structure of distal genitalia (**54**), transverse sections of medial epiphallus (**55**) and apical penial papilla (**56**) of *Monacha pantanellii* from Carsoli [Car] (FGC 41651).

**Figures 57–59.**
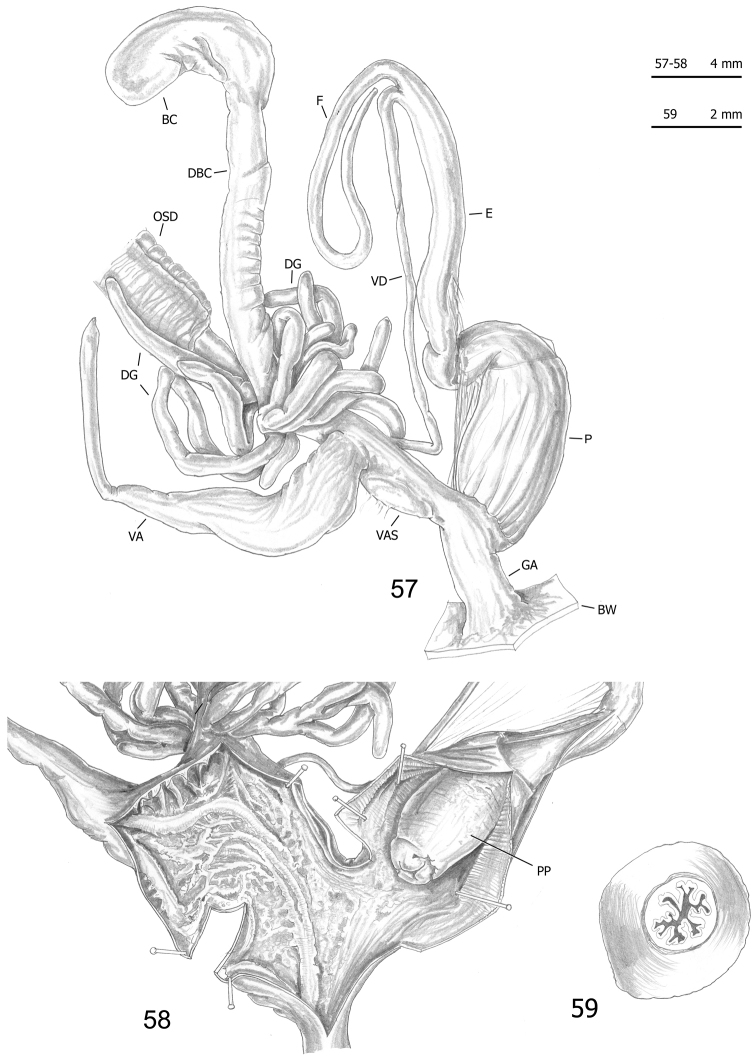
Genitalia (proximal parts excluded) (**57**), internal structure of distal genitalia (**58**) and transverse section of apical penial papilla (**59**) of *Monacha pantanellii* from Lago del Turano [Tur2] (FGC 41654).

**Figures 60–63.**
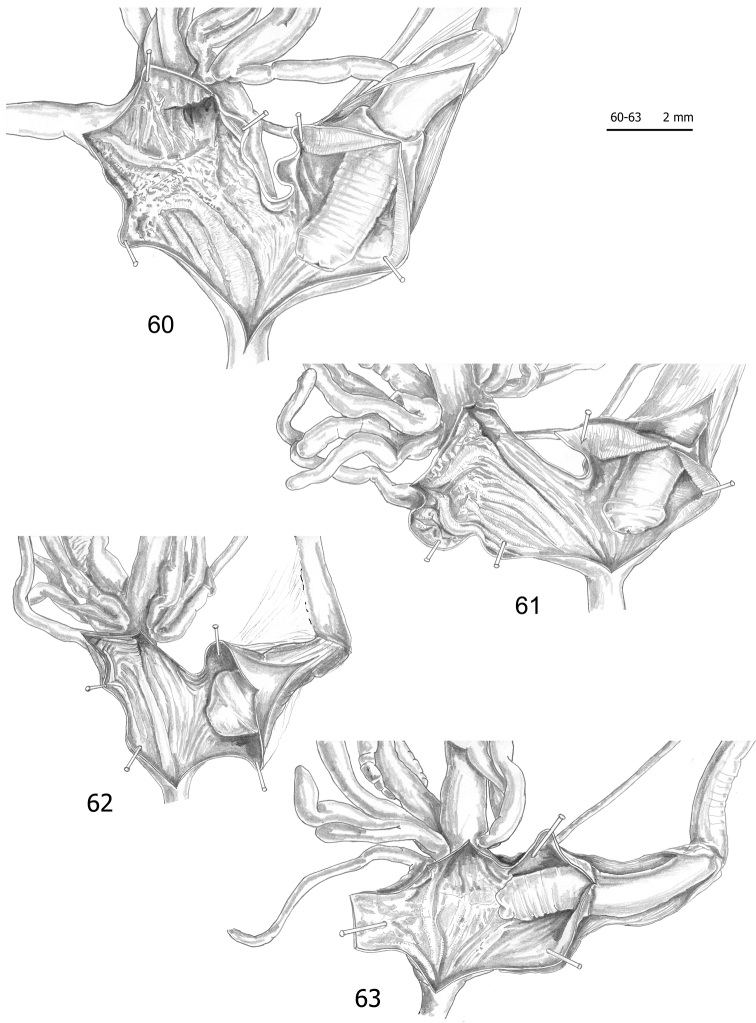
Internal structure of distal genitalia of *Monacha pantanellii* from Valle dell’Aniene, Roccagiovine [Ani] (FGC 42974) (**60**), Via Salaria, Ornaro Alto [Alt] (FGC 41553) (**61**), Turania [Tur1] (FGC 42971) (**62**) and road to Montenero Sabino [Sab] (FGC 41552) (**63**).

**Figures 64, 65.**
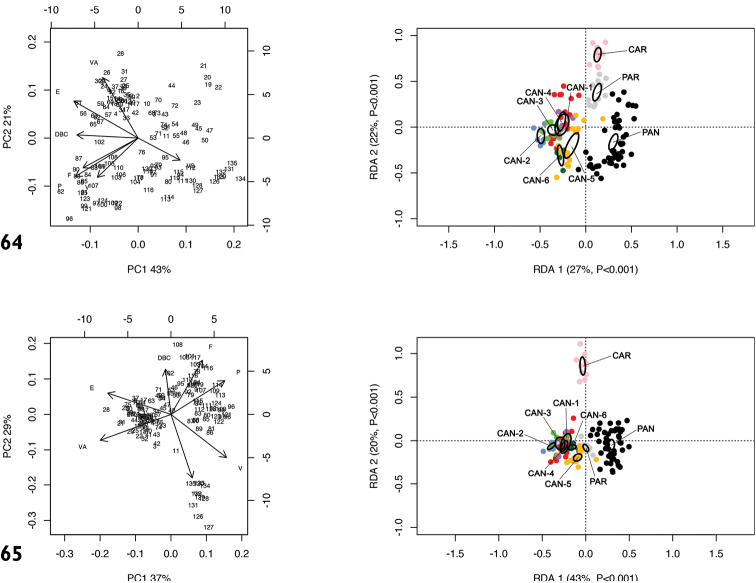
Principal component analysis (PCA) and Redundancy analysis (RDA) with species or molecular lineage constraint applied to the original genital matrix (**64**) and shape-related Z-matrix (**65**).

RDA with species or molecular lineage constraint on the shape and size matrix (Fig. 64) showed that RDA 1 (27%, P < 0.001) separated *M. cantiana* s. l. (CAN-1, CAN-2, CAN-3, CAN-4, CAN-5 and CAN-6) from PAN, PAR and CAR. The preliminary classic PCA revealed size as the first major source of morphological variation, since PC1 (43%) accounted for VS vs. all the other variables. On the contrary, RDA 2 (22%, P < 0.001) separated CAN-5, CAN-6 and PAN from CAR and PAR. The group CAN-1, CAN-2, CAN-3 and CAN-4 was in intermediate position. In that regard, PC2 (20%) accounted for a contrast between E and VA vs. F, P, V and VS.

RDA with species or molecular lineage constraint on the shape (Z) matrix (Fig. 65) showed that RDA 1 (43%, P < 0.001) separated PAN from the group CAN-1, CAN-2, CAN-3, CAN-4 and CAN-6, with CAN-5, PAR and CAR in intermediate position, and that RDA 2 (20%, P < 0.001) separated CAR from all the others. Shape-related PCA indicated that VA and E vs. V and P were the principal shape determinants on PC1 and VS and V vs. DBC and F on PC2. In the latter case, removing the size effect altered the overall relationship patterns.

**Figure 66.**
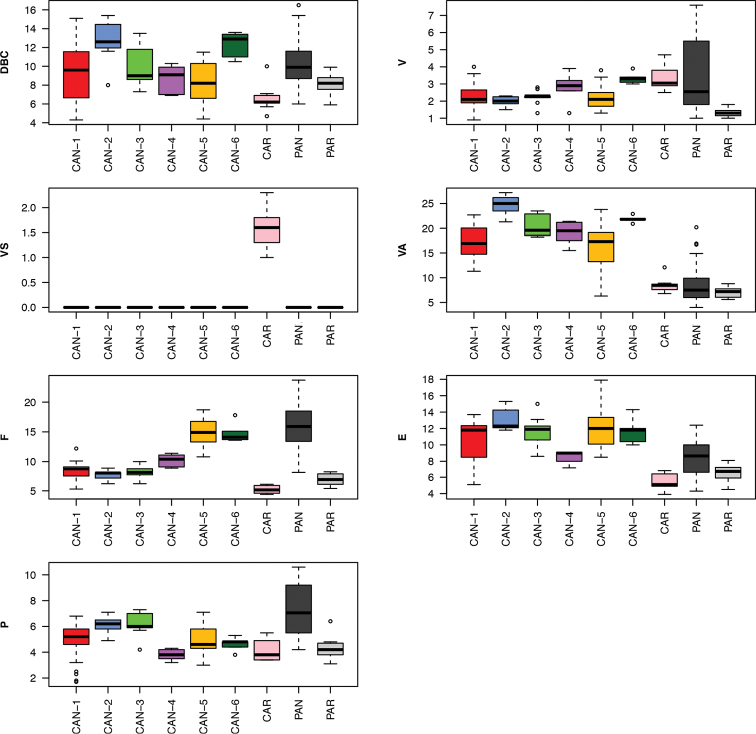
Box plots for genital characters of the ten *Monacha* species or molecular lineages investigated. The lower and upper limits of the rectangular boxes indicate the 25^th^ to 75^th^ percentile range, and the horizontal line within the boxes is the median (50^th^ percentile).

Box plots (Fig. 66) for anatomical characters showed that VA, F and P have the best discriminating value in distinguishing PAN: they distinguished 6 (VA) and 5 (F and P) species or molecular lineage pairs, respectively, according to Dunn’s test with Benjamini-Hochberg adjustment (α = 0.01), followed by E and V with four and three species or molecular lineage pairs, respectively (Table 6). The most recognisable pairs were PAN vs. CAR and PAN vs. CAN-1 (four significant characters), PAN vs. CAN-2, PAN vs. CAN-3, PAN vs. CAN-5 and PAN vs. PAR (3 significant characters). Only two characters significantly distinguished PAN vs. CAN-4 and only one PAN vs. CAN-6 (Table 6). Anatomical characters have high discriminating value as testified by very low p values after Dunn’s test: in most cases (19 of 22) P < 0.001 (Table 6).

**Figures 67, 68.**
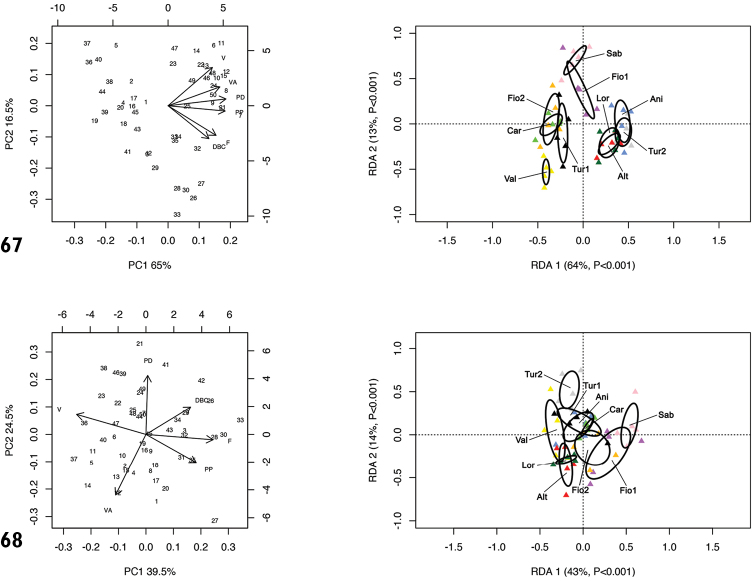
Principal component analysis (PCA) and Redundancy analysis (RDA) with population constraint applied to the original genital matrix (**67**) and shape-related Z-matrix (**68**) of specimens of *Monacha pantanellii*.

RDA with population constraint on the shape and size matrix (Fig. 67) showed that RDA 1 (64%, P < 0.001) separated populations Carsoli [Car], Monte Fionchi, Torrecola [Fio2], Turania [Tur1] and Vallonina [Val] from populations Via Salaria, Ornaro Alto [Alt], Valle dell’Aniene [Ani], Via Salaria, Poggio San Lorenzo [Lor] and Lago del Turano [Tur2], with Monte Fionchi, summit [Fio1] and Montero Sabino [Sab] in intermediate position. The preliminary classic PCA revealed size as the first major source of morphological variation, since PC1 (65%) was a positive combination of all variables. On the contrary, RDA 2 (13%, P < 0.001) separated population Val from populations Sab and Fio1, with all the other populations (Car, Fio2, Tur1, Alt, Ani, Lor and Tur2) in intermediate position. In that regard, PC2 (16.5%) accounted for a contrast between V and VA vs. F and DBC.

RDA on the shape (Z) matrix (Fig. 68) showed a less clear separation between populations. RDA 1 (43%, P < 0.001) separated the population Sab from the group of populations Tur2, Val, Alt and Lor, with Ani, Fio1, Fio2 and Car in intermediate position. Shape-related PCA indicated that V vs. F were the two principal shape determinants on PC1 (39.5%). RDA 2 (14%, P < 0.001) separated Tur2 from Alt, Lor and Fio1, with all the other populations in a more or less intermediate position. In that regard, PC2 (24.5%) accounted for a contrast between PD and VA.

## Discussion

Molecular analysis of nucleotide sequences obtained from specimens originating from ten populations occurring in the grasslands of the central Apennines suggests that these populations represent a different species from other Italian *M. cantiana* s. l. lineages (CAN-1, CAN-2, CAN-3, CAN-5, CAN-6) and *Monacha* species (*M. cartusiana* and *M. parumcincta*), populations of which were previously subject to molecular analysis (Pieńkowska et al. 2018a, 2019a, 2019b). In each of the phylogenetic trees, i.e., ML of concatenated sequences for mitochondrial COI+16S rDNA (Fig. 2) and nuclear ITS2+H3 (Fig. 3) gene fragments as well as the BI tree of concatenated sequences COI+16S rDNA+ITS2+H3 (Fig. 4), sequences from these ten populations created well separated monophyletic clades. Two of these populations represent species described in the past: Monte Fionchi, Summit [Fio1]: *Helix pantanellii* De Stefani, 1879; Vallonina [Val]: *Monacha ruffoi* Giusti, 1973. Molecular analysis confirmed the validity of the species described by Giusti (1973) from the Reatini Mountains, although an older discarded name, introduced by De Stefani (1879), turned out to be available for it.

The range of K2P genetic distances between COI sequences obtained from the ten populations of *M. pantanellii* was 0.2–6.7% (Table 5). We previously found a similar range of K2P distances within populations of *M. cantiana* s. l. CAN-1/CAN-2 (0.2–5.3%; Pieńkowska et al. 2018a, 2019b), *M. cartusiana* (0.0–3.3%; Pieńkowska et al. 2015, 2016, 2018b), *M. parumcincta* (0.2–4.6%; Pieńkowska et al. 2018a, 2019b) and *M. claustralis* (0.0–5.7%; Pieńkowska et al. 2015, 2016, 2018b). It is worth noting that this K2P distance range was even narrower (0.2–4.5%) if we considered all but three of 53 the COI sequences obtained from *M. pantanellii* specimens. The three COI sequences excluded were found in single (one or two) specimens of populations from Carsoli [Car], Valle del Turano [Tur1] and Vallonina [Val], however COI sequences obtained from the other specimens of these populations were more similar to others found in *M. pantanellii*. This suggests higher intra-population variation within these three populations, which may prove speciation seen in a rapidly evolving mitochondrial genome (Thomaz et al. 1996; Remigio and Hebert 2003).

The conclusion that ten populations from the central Apennines form a different species is supported by the analysis of K2P genetic distances of COI sequences (Table 5). Although the utility of the 3% barcode threshold as a marker for species distinction, applied in the so-called “barcode method” based on COI sequences (Hebert et al. 2003a, 2003b, 2018; Pentinsaari et al. 2020), is disputable (Davison et al. 2009; Sauer and Hausdorf 2010, 2012; Köhler and Johnson 2012; Batomalaque et al. 2019; Koch et al. 2020), COI sequences have been used to analyse taxonomic problems in different gastropod families (e.g., Remigio and Hebert 2003; Elejalde et al. 2008; Duda et al. 2011; Breugelmans et al. 2013; Proćków et al. 2013, 2019; Čandek and Kuntner 2015; Walther et al. 2016; Kruckenhauser et al. 2017; Galan et al. 2018; Gladstone et al. 2019; Harl et al. 2019; Kneubühler et al. 2019; Bamberger et al. 2020). They were also useful in our previous studies on *Monacha* species (Pieńkowska et al. 2015, 2018a, 2019a, 2019b). Indeed, we have always emphasised that phylogenetic analysis cannot be based on a single gene locus but should combine several mitochondrial and nuclear genes (Pieńkowska et al. 2015, 2018a, 2019a, 2019b). Note that the conclusion that ten populations are distinct from other *Monacha* species at species level is not only supported by the analysis of COI sequences, but also of 16S rDNA, ITS2, and H3.

Moreover, we have always stressed (Pieńkowska et al. 2015, 2018a, 2019a, 2019b) that molecular features alone are insufficient to define species but need to be supported by morphological (shell and anatomy) features. Inconsistency between molecular and morphological features may occur among snail populations or species (Cameron 1992; Cameron et al. 1996; Sauer and Hausdorf 2012; Falniowski et al. 2020), because according to the concept of morphostatic evolution (Gittenberger 1991; Davis 1992; Koch et al. 2020) speciation may be reflected earlier in molecular than in morphological features.

It is not possible to distinguish *M. pantanellii* from the lineages of the *M. cantiana* group on the basis of shell characters, perhaps with the exception of CAN-6 (see Figs 32–34; Table 6). However, this may be biased by the fact that only one population of this lineage was available for study (Pieńkowska et al. 2019b). With regard to the other two species examined by comparison, *M. cartusiana* and *M. parumcincta*, the analysis found that distinguishing *M. pantanellii* from the former is difficult (only two characters have discriminating value), but from the latter is easy (eight characters have discriminating value). Anyway, these species are readily distinguished by colour pattern. *M. cartusiana* has a smoother more glossy shell, usually whitish, often with sharp milky-white subsutural and peripheral bands, intensely reddish-brown peristome, externally bordered by a ring of bright milky white. *M. parumcincta* has a shell similar to that of *M. pantanellii*, but less glossy and more opaque, sometimes with paler peripheral and subsutural bands and brownish peristome, externally bordered by a pale whitish ring.

The distinction of *M. pantanellii* based on anatomical characters is clear from the lineages of the *M. cantiana* group and the other two species examined by comparison, *M. cartusiana* and *M. parumcincta*. However, contrary to the situation with shell characters, CAN-6 is the lineage least distinct from *M. pantanellii*: again, the few specimens available may have biased the result. The analysis confirmed the high discriminating value of the vaginal appendix which distinguishes *M. pantanellii* from all the lineages of the *M. cantiana* group and *M. cartusiana*. The penis and flagellum are also important because they significantly distinguish *M. pantanellii* from five other species or molecular lineages (Table 6).

Other anatomical features that distinguish *M. pantanellii* from the *M. cantiana* group, *M. cartusiana* and *M. parumcincta* were not included in the analysis, since it is impossible to quantify them. They are the insertion of the vaginal appendix, the shape of the vaginal appendix, and the section of the penial papilla (Table 7).

**Table 7.** Other anatomical features distinguishing *Monacha* species.

Characters	*M. pantanellii*	*M. cantiana* group	*M. cartusiana*	*M. parumcincta*
**insertion of VA**	vaginal	atrial	vaginal	atrial
**shape of VA**	usually short and slender, calibre almost constant; however, in two populations it is long or very long with proximal portion (ca. half its length or more) very enlarged and distal portion slender	long or very long, not slender nor enlarged, calibre initially large then progressively tapered; sometimes with variably evident basal sac	long or very long with proximal portion (ca. half its length or less) enlarged and distal portion slender	usually short and enlarged, calibre almost constant
**PP**	with thick external walls and narrow space between external walls and central duct; central duct circular in transverse section, usually rather small in diameter, not joined by strips to external walls and with its lumen almost totally filled by large pleats	with thick external walls, and narrow to wide space between external walls and central duct; central duct circular in transverse section, usually rather large in diameter, joined by strips to external walls and with its lumen not filled by large pleats	with thick external walls and narrow to wide space between external walls and circular central duct; central duct circular in transverse section, usually medium-sized in diameter, not joined by strips to external walls and with its lumen almost totally filled by large pleats	with thin external walls and narrow space between external walls and central duct; central duct horseshoe-shaped in transverse section, large in diameter, not joined by strips to external walls and with its lumen apparently not filled by pleats
**References**	Giusti (1973: figs 26A, B), this paper (Figs 38–64)	Pieńkowska et al. (2018a: figs 20–50; 2019a: figs 2–3; 2019b: figs 19–41)	Giusti and Manganelli (1987: figs 1A–G), Pieńkowska et al. (2015: figs 11–12, 15–16, 18–21)	Pieńkowska et al. (2018a: figs 51–59)

Intraspecific variability in *M. pantanellii* is high and concerns both shell and genitalia. Inter-population shell variability mainly affects the size features: some populations are distinguished by reduced size, notably the one from Carsoli [Car] (Figs 30, 31) and the slightly larger populations from Turania [Tur1] (Figs 11–14) and Vallonina [Val] (Figs 21, 22). This pattern was confirmed by RDA on the original shell matrix (Fig. 32) and by its disappearance when the size effect was removed (Fig. 33). Anatomically, these populations agree very well with the characters typical of the species (e.g., VA, PD, F) suggesting that shell size has no phylogenetic signal and cannot be used to support taxonomic distinctions. We can hypothesize that it depends on local conditions of drought, food availability and lack of refuges.

Intra-population shell variability is smaller, but the variation of UD from Via Salaria, Ornaro Alto [Alt] is notable (0.9–2.4 mm) including almost the extremes of the range (Figs 18–20).

Inter-population genital variability is more intricate although the size effect is again evident: RDA 1 (Fig. 35) separates the populations of smaller size, i.e., those from Carsoli [Car], Monte Fionchi, Torrecola [Fio2], Turania [Tur1] and Vallonina [Val], from those of larger size, namely Via Salaria, Ornaro Alto [Alt], Valle dell’Aniene, Roccagiovine [Ani], Via Salaria, Poggio San Lorenzo (Lor] and Lago del Turano [Tur2]. When the size effect is removed (Fig. 36) some patterns persist, albeit less clear because conflicting variables are involved. Inter-population genital variability concerns all anatomical sections but is higher in V and VA (as shown by PCA). The former (V) is very short in Montenero Sabino [Sab] (Fig. 63), Monte Fionchi, Torrecola [Fio2] (Fig. 41) and Carsoli [Car] populations (Fig. 53) and long in those from Via Salaria, Ornaro Alto [Alt] (Fig. 61), Via Salaria, Poggio San Lorenzo [Lor] (not shown), Valle dell’Aniene, Roccagiovine [Ani] (Fig. 49) and Lago del Turano [Tur2] (Fig. 57). The latter (VA) is usually short but is long in Valle dell’Aniene, Roccagiovine [Ani] (Fig. 49) and very long in Lago del Turano [Tur2] populations (Fig. 57), where however intra-population range is wide.

According to RDA on the shape (Z) matrix, some of the most divergent populations are those from Montenero Sabino [Sab] and Lago del Turano [Tur2], which fall at the extremes of the ordination figure (Fig. 68).

This revision is the first result of research on the *Monacha* species living in the mountain grasslands of the central Apennines. It confirms the validity of the species described by Giusti (1973) from the Reatini Mountains, though an older discarded name, introduced by De Stefani (1879), turned out to be available for it.

It is evident from the above discussion that the species of *Monacha* and the lineages of *M. cantiana* s. l. can only occasionally be recognised morphologically and are also subject to significant inter- and intra-population variability. In this situation, revision based on type material consisting of shells may be not decisive. We therefore took an overall approach that considers shell, genital and molecular evidence to establish a reliable taxonomic setting. Only a multidisciplinary investigation of populations from the type locality, matching type specimens, can clarify the identity of old established *Monacha* taxa. This what we tried to do, although it was made difficult by the fact that the type locality was not always reported in a detailed way. Luckily this was not the case of the species described by De Stefani (1879). Thus, the investigation of specimens from the type locality, the summit of Monte Fionchi near Spoleto in Umbria, enabled us to ascertain that they have the same anatomical features as *M. ruffoi*. Conspecificity of the topotypical populations of *M. pantanellii* and *M. ruffoi* is also strongly supported by molecular analysis. Consequently, the latter has to be regarded as a junior synonym of De Stefani’s species.

Since *M. pantanellii* is a *Monacha* species with distinctive anatomical features, we checked all the material accessible to us. This enabled us to find other populations of the species, some from the Reatini Mountains, where they were collected by one of us in the 1960s during field work, some from other more northern mountain ranges (Table 3).

Regarding relationships of *M. pantanellii* with other taxa described or reported from the central Apennines, research is underway. So far we can only reveal that they belong to lineages different from this species and the *M. cantiana* group.

## Redescription of *Monacha pantanellii* (De Stefani, 1879)

### Monacha pantanellii

Taxon classificationAnimaliaStylommatophoraHygromiidae

(De Stefani, 1879)

9F28FA85-571C-556E-9394-3A5E8A6CE89E

Figures 5–14 , 15–22 , 23–31 , 37–40 , 41–44 , 45–48 , 49–52 , 53–56 , 57–59 , 60–63

Helix pantanellii De Stefani, 1879: 40–41.
Monacha ruffoi Giusti, 1973: 533–537, pl. 6.

#### Diagnosis.

A species of *Monacha* (s. str.) (according to the subgeneric division proposed by Neiber and Hausdorf 2017) with vaginal appendix usually short and slender (having shape and size of a digitiform gland) inserted at mid vagina; proximal vaginal sac absent; penial flagellum long to very long; penial papilla with narrow space between external walls and central duct; central duct circular in transverse section, usually rather small in diameter, not joined by strips to external walls and with its lumen almost totally filled by large pleats.

#### Redescription.

*Shell* (Figs 5–31) dextral, sub-globose to globose, small to medium in size, variable in colour, sometimes (when colour is brownish yellow) with paler subsutural and peripheral bands, with 5¼–6 slightly convex whorls separated by superficial sutures; aperture slightly prosocline, round to oval; peristome not reflected, thickened, with variably evident whitish callous rim lining the outer margin; umbilicus open, very small to small; protoconch and teleoconch smooth, with very faint scattered collabral growth lines. Shell dimensions: H: 10.3 ± 1.5 mm; D: 16.2 ± 2.3 mm (n = 45).

*Radula* not examined.

*Female distal genitalia* (Figs 37, 38, 41, 42, 45, 46, 49, 50, 53, 54, 57, 58, 60–63; Table 7) include free oviduct, bursa copulatrix and its duct, digitiform glands, vagina and vaginal appendix. Free oviduct short and variably wide. Bursa copulatrix bean-like or pyriform with long wide duct. Vagina short to long and wide. Digitiform glands disposed on opposite sides of vagina in two groups of 1–3 tufts, each with 1–3 units. Vaginal appendix usually short (having shape and size of a digitiform gland) and inserted approximately half-way along the vagina.

*Male distal genitalia* (Figs 37–63, Table 7) include vas deferens, flagellum, epiphallus and penis. Vas deferens very long and very slender. Flagellum long to very long and slender. Epiphallus long to very long and wide. Penis short and wide, enveloped by thin sheath, consisting of proximal portion (from start of penial sheath to base of penial papilla) and distal portion (from base of penial papilla to genital atrium). Penial papilla variable in shape (perhaps due to pre-mortem stress or spirit fixation), with apical opening, thick external walls and narrow space between external walls and central duct; central duct circular in section, usually rather small in diameter, not joined by strips to external walls of penial papilla and with its lumen almost totally filled with large pleats.

Genital atrium large, receiving vagina and penis, internally smooth or with variably developed longitudinal pleats.

#### Type locality.

“Sulla cima del Monte Fionghi al sud di Spoleto a circa mille metri sul livello del mare “, i.e., on the summit of Monte Fionchi, south of Spoleto, at an altitude of ca. 1000 m (municipality of Spoleto, province of Perugia), UTM references 32T UH 1726, Lat and Long: 42°40.455'N, 12°46.340'E.

#### Type material.

Probably lost.

#### Topotype sequences.

Sequences obtained from individuals from the type locality of *M. pantanellii* are designated as typical for this species: COI – MT380011–MT380018, 16S rDNA – MT376031–MT376039, ITS2 – MT376088–MT376094, H3 – MT385776–MT385785.

#### Etymology.

Named after Dante Pantanelli (1844–1913), Italian palaeontologist and geologist at the University of Modena. He published many papers on Miocene and Pliocene molluscs, some of which were co-authored by his friend Carlo De Stefani (1851–1924). He was also the secretary of the Italian Malacological Society and the editor of the Bullettino della Società Malacologica Italiana for many years (Manganelli et al. 2017, with references).

Giusti’s species was named after Sandro Ruffo (1915–2010), a major Italian twentieth-century zoologist and director of the Museo Civico di Storia Naturale di Verona for many years (Latella 2011).

#### Distribution.

Endemic to Umbria-Marche Apennines and Latium Sub-Apennines. It occurs from the Apennines of Gualdo Tadino in the north to the Aniene and Turano valleys in the south.

#### Ecology.

Mesophile species living among grass in open habitats such as grasslands, pastures, forest edges and clearings in hill and mountain areas.

#### Conservation.

Apparently common and widespread species within its range, but in some sites (e.g., Vallonina) it was no longer found during a field survey in the summer of 2019. Like other mesophilic species it could be sensitive to global warming.

#### Remarks.

This species was distinguished from *Monacha cantiana* on the basis of a few shell characters (“more depressed, more fragile and paler shell, with fine growth lines, less rounded opening and deeper umbilicus”) and was disregarded by its author as an “extreme variety” of the former. Subsequently it was only reported in two catalogues by Westerlund (1889: 95) and Pilsbry (1895: 266) so that when Alzona prepared the catalogue of Italian non-marine malacofauna, they included it as a doubtful species (Alzona 1971: 183).

On the contrary, our analysis showed that it matches a valid species, currently known as *Monacha ruffoi*, described from the Reatini mountains by Giusti (1973) as a *Monacha* species with a shell resembling that of *cantiana*, but with a much smaller vaginal appendix.

This is an unexpected result: indeed, De Stefani’s species is one of thousands of mollusc species established since the second half of the nineteenth century on the basis of very few shell features of no diagnostic value due to dramatic intra- and inter-population variability. In describing thousands of species and varieties, past authors hit on some that remained valid.

## Supplementary Material

XML Treatment for Monacha pantanellii
